# Promoting gender equity in very young adolescents: targeting a window of opportunity for social emotional learning and identity development

**DOI:** 10.1186/s12889-021-12278-3

**Published:** 2021-12-19

**Authors:** Megan Cherewick, Sarah Lebu, Christine Su, Lisa Richards, Prosper F. Njau, Ronald E. Dahl

**Affiliations:** 1 Department of Community and Behavioral Health, Colorado School of Public Health, University of Colorado Anschutz, 13001 E 17th Pl, Room B119, Aurora, CO 80045 USA; 2 Institute of Human Development, University of California Berkeley, 2121 Berkeley Way West, Berkeley, CA 94720 USA; 3 Health for a Prosperous Nation, P.O. Box 13650, Dar es Salaam, Tanzania

**Keywords:** Adolescence, gender equity, social emotional learning, gender norms and attitudes

## Abstract

**Background:**

The transition from childhood to adolescence is a uniquely sensitive period for social and emotional learning in the trajectory of human development. This transition is characterized by rapid physical growth, sexual maturation, cognitive and behavioral changes and dynamic changes in social relationships. This pivotal transition provides a window of opportunity for social emotional learning that can shape early adolescent identity formation and gender norms, beliefs and behaviors. The objective of this study is to evaluate the potential of a social emotional learning intervention for very young adolescents (VYAs) to improve social emotional mindsets and skills.

**Methods:**

Discover Learning is a social emotional learning intervention designed for VYAs (10-11 years of age) to support development of social emotional mindsets and skills from four primary schools in Dar es Salaam, Tanzania. The intervention delivered three different packages of learning experiences to three arms of the study. 528 VYAs were randomized to each of the three study arms (A-Content learning, B-Content learning and reflection, and C-Content learning, reflection and experiential practice). A quantitative survey was administered to all participants before and after the intervention to capture changes in social emotional mindsets and skills. A discrete choice experiment measured changes in gender norms, beliefs and behaviors.

**Results:**

528 VYAs were included in the analysis. Participants in all three arms of the study demonstrated significant improvements in social emotional mindsets and skills outcomes (generosity, curiosity, growth mindset, persistence, purpose and teamwork). However, Group C (who received experiential social learning opportunities in small, mixed-gender groups and a parent and community learning components demonstrated larger treatment effects on key outcomes in comparison to Groups A and B. Results indicate Group C participants had greater change in gender equity outcomes (OR = 1.69, p = <0.001) compared to Group A (OR = 1.30, p = <0.001) and Group B (OR = 1.23, p = 0.004).

**Conclusion:**

These findings provide evidence that social emotional learning interventions targeting VYAs can improve social emotional mindsets and skills and gender equity outcomes. The findings indicate the importance of *experiential* learning activities in mixed-gender groups during the unique developmental window of early adolescence. The study also provides support for the inclusion of parental/caregiver and community engagement in programs designed for VYAs.

**Trial registration:**

Retrospectively registered on July 7^th^, 2020. NCT0445807

**Supplementary Information:**

The online version contains supplementary material available at 10.1186/s12889-021-12278-3.

## Background

The Lancet Youth Commission Report highlighted the need for investment in the largest generation of 10- to 24-year-olds in human history [1]. There were approximately 1.24 billion adolescents representing 16% of the global population in 2018 [2]. Adolescence is a period of vulnerability where physical and mental health problems emerge and can persist into adulthood [3]. Increases in the incidence of accidents, suicide, homicide, mental disorders, substance use, eating disorders, sexually transmitted diseases and unintended pregnancy pose risks to health trajectories [1]. Interventions to address these risks often target older adolescents [15–19] and miss an opportunity to impact health trajectories that begin during early adolescence [4, 5]. More recent research has recognized that very young adolescence is a unique period of development to promote healthy trajectories before social, emotional, cognitive and behavioral vulnerabilities intensify [6, 7].

Very young adolescence, before the onset of puberty presents a key opportunity to promote positive health and well-being trajectories that can have an enduring impact throughout life [8]. Changes during this period include rapid physical growth and brain development, sexual maturation and changes in cognitive, social, emotional, psychological and behavioral processes [8]. This dynamic developmental window is characterized by a unique combination of stability and plasticity in developing neural networks that can amplify the salience of experiential and social emotional learning [9]. During this distinctive maturational period, adolescents are particularly sensitive to learning opportunities that empower them to navigate an increasingly complex social world.

Research on adolescent developmental trajectories have highlighted the risks for adverse health outcomes that begin during adolescence and can persist into adulthood [10]. Less research has focused on the opportunity to promote positive health trajectories during this pivotal window of development— not only to protect adolescents from risks in later adolescence but also to support social emotional mindsets and skills that lead to healthy identity development. Social emotional mindsets can shape motivational proclivities that are particularly relevant not only to identity development, but also to adapting successfully to rapidly changing social contexts. Social emotional skills capture changes in adolescents’ relational skills and capacities to respond to challenges and opportunities in their social world such as teamwork, empathy, and gender equitable behaviors [6].

Findings from the developmental science of adolescence indicate that the onset of puberty is associated with important neurological changes that impact learning, including increases in motivational learning, sensation-seeking, novelty and a sensitivity for peer admiration. Adolescent behavior is associated with adaptive developmental proclivities to explore and understand one’s social world, including social roles, social hierarchies, issues of social acceptance, admiration, and learning to establish individual identity [11]. Early experiential learning during adolescence—particularly in the realm of social emotional learning about self/other and social relationships shape the development of an individual’s identity, including mindsets, skills, and behavioral proclivities [12, 13]. Shaping these mindsets, skills, and proclivities during a sensitive window of learning can have an impact on proximal and distal health outcomes, as well as social relationships and academic attainment [14].

More equitable gender norms, beliefs and behaviors can impact sexual and reproductive health outcomes, protect against vulnerability to gender-based discrimination and violence and reduce risk for gender inequities in mental health disorders and well-being. Patterns of social behavior, emotional development and identity formation during early adolescence can impact gender-related experiences and associated health outcomes. Pubertal maturation is a formational time—in part because of the rapid physical changes that occur with sexual maturation, and in part because local social and cultural influences shape gendered experiences. For example, as girls go through puberty, they are often treated differently in their social context and are increasingly restricted from participating in activities outside of the house or mixed-gender activities.

In addition to the opportunity to shape gender norms, beliefs and behaviors for very young adolescents, there are a broad range of similar opportunities—such as acquiring knowledge, skills, and motivational proclivities in the areas of *growth mindset*, *curiosity, generosity, persistence, purpose, and teamwork* that can increase adaptive capacities for social and academic success in young adolescents. These social emotional mindsets and skills are particularly relevant for youth growing up in rapidly changing low- and middle-income countries (LMICs), where academic success and technology are altering the landscape of opportunities and vulnerabilities for youth.

A growing body of evidence indicates that adolescents between the ages of 10-14 actively build their identities, establish behaviors, gain social knowledge, and shape their values and beliefs systems [8, 15]. There is extensive literature that explores interventions targeting social emotional outcomes, including gender norms and beliefs among adolescents. Each of these studies focus on domains of social emotional learning and gender equity, such as sexual and reproductive health [16], role of parents and community [17], gender-based violence [18], and health and well-being [19, 20]. The Global Early Adolescent Study (GEAS) conducted with 10–14-year-olds in the Democratic Republic of Congo, found that mixed gender group sessions were a valuable implementation strategy to maximize learning positive gender norms and beliefs [21]. In rural India, a study showed that when the same school-based gender transformative programming was delivered to younger (aged 13–14 years) and older (aged 15–19 years) boys, the impact of the program was significantly greater for those who were younger [22, 23].

## Adolescence in Tanzania

In low-resource contexts, adolescents often experience more concentrated stress and adverse life experiences than in higher resource contexts. Research and practice has increasingly focused on the need to target very young adolescents to improve their health trajectories in low-resource contexts and attenuate risks associated with adverse life experiences [24]. In low-income countries, evidence suggests that programs that target VYAs can have long-term impact such as decreasing the spread of HIV/AIDS [25], unwanted pregnancies [26] and improving health and well-being [27]. Tanzania’s youth population has risen with 47% of the population under the age of 15 years [28, 29]. The total adolescent population is expected to double by 2055 [2]. This youth ‘bulge’ is the result of improvements in reducing early child morbidity and mortality; however, there is an associated increase in morbidity and mortality from preventable causes during adolescence and adulthood [1].

Results of a child poverty study in Tanzania found that 74% of children in Tanzania are affected by multidimensional poverty and 29% live in households below the monetary poverty line [30]. The Global Out-Of-School Children Study conducted in Tanzania estimated that 3.5 million school-aged children and adolescents were not in enrolled in school in 2017 [31]. Tanzania’s Demographic Health Survey found that adolescent girls who were not in school were five times more likely to have children, with 27% of girls ages 15-19 already pregnant with their first child [28]. If Tanzania makes strategic investments in adolescents, it can benefit from a workforce with greater educational attainment and economic opportunities.

Tanzania has made progress to improve access to education with the introduction of free primary education in 2001. As a result, enrollment in secondary education tripled for girls and quadrupled for boys between 2004 and 2010 [32]. Evidence from the Global Out of School Report for Tanzania found that attendance rates increase with age, with enrollment peaking at the age of 11 with notable reductions in enrollment at the age of 13 years [31]. While there has been an increase in school enrollment, little has been done to expand school infrastructure and resources to accommodate this increase. The ratio of pupil-to-qualified teachers rose from 1:57 in 2007 to 1:66 in 2016 [31, 33]. Schools face shortages in books, classroom furniture and facilities such as toilets and potable water. School climate affects adolescent learning and development and in Tanzania, over 50% of students report being mistreated by a teacher [31]. While the introduction of free primary education has resulted in higher secondary education enrollment, there are important equity gaps. Transition to secondary school is an important measure of academic success and approximately 21% of boys joining secondary schools compared to 16% girls [34]. Children from low-income households have increased enrollment in primary school but continue to be disadvantaged. Girls from the lowest income quintile are twice as likely to drop out compared to girls from higher income quintiles [31].

Gender disparities in enrollment patterns and educational outcomes have led researchers to focus on the role of gender, sexual and reproductive health and education outcomes. In Tanzania, it is estimated that 31% of women who are between 20-24 years of age were married before their 18^th^ birthday [35]. In 2016, 1 in 4 adolescent girls between the ages of 15-19 had begun childbearing, reflecting a 4% increase in teenage pregnancy since 2010 [36]. Cultural norms around gender and sexuality contribute to unfavorable sexual and reproductive health outcomes for girls. Differences in normative gender roles manifest in a variety of ways such as boys being allowed more freedom outside the house, whereas girls spend more time in the home and have greater responsibility to complete household chores [37, 38]. A cultural prototype of a chaste female student is highly valued in Tanzania and at the same time, girls at risk for sexual exploitation. Transactional sex increases vulnerability to gender based violence, unwanted pregnancy and sexually transmitted diseases.

Investing in programs for VYAs that include gender transformative content can help address gender inequities. Learning in mixed-gender groups before the onset of puberty and sexual debut provides opportunities to promote healthy gender norms, beliefs and behaviors for both boys and girls. Previous studies targeting sexual and reproductive health of adolescents in Tanzania often focus on later adolescence (ages 15-19) and miss a critical opportunity to shape gender norms, beliefs and behaviors that can enhance healthy development of girls and protect against risks to sexual and reproductive health that emerge during later adolescence.

## Technology as a novel and exciting learning tool

Advances in access and acceptability of technology offer an increasingly important opportunity for social, emotional and identity learning. By 2017, 80% of households reported use of mobile phones in Tanzania [39]. The changing technological landscape in low-income countries is a promising opportunity to advance learning and address inequities in overburdened, under-resourced education systems [40]. Policy makers in low-resource contexts are increasingly open to incorporating technological tools for education [40]. Tanzania’s National Information and Communication Technology (ICT) Policy of 2003 acknowledges that ICT offers a new opportunity to improve education in all areas [41]. Adolescents are often early adopters of technology and are motivated to learn and master technology. The natural motivation of adolescents to explore, discover and master novel and stimulating environments is an opportunity to deliver a high impact intervention through technological platforms.

The objective of Discover Learning *(Discover)* was to test a social emotional learning intervention designed for VYAs to promote positive, gender transformative, social, emotional mindsets and skills to promote healthy developmental trajectories during the transition from childhood into adolescence. The primary aims of *Discover* were 1) To test the effectiveness of providing learning opportunities that focus on social emotional mindsets and skills including curiosity, generosity, persistence, purpose, growth mindset and teamwork; 2) to evaluate the use of digital technology for social, emotional and identity learning, and 3) to identify high-impact components of the intervention with the potential to be scaled in Tanzania and in similar low-resource contexts. *Discover* is a strategic partnership between University of California Berkeley, Health for a Prosperous Nation (HPON), Camara Education, Save the Children, Dalberg Consulting and Ubongo Kids.

## Methods

### Study design

This study is a three-arm comparative effectiveness trial evaluating *Discover* [17, 42], an intervention designed to support learning social emotional mindsets and skills among very young adolescents (10-11 years old). Learning content included 6 modules targeting growth mindset, curiosity, generosity, persistence, purpose, and teamwork. Modules included content created in collaboration with Ubongo Kids, a Tanzania-based organization that develops engaging and locally relevant digital content for children in Africa. Each video featured a 10–15-minute episode of animated cartoon characters with storylines and songs for each of the 6 social emotional mindset and skill content areas. The comparative effectiveness trial included three study arms (Group A, B and C). Each study arm received a different set of learning opportunities including 1) Viewing *Ubongo Kids* video content on social emotional mindsets and skills, 2) reflective discussion with peers, 3) experiential learning activities in small mixed-gender groups, 4) learning experiences exploring and mastering technology, 5) learning experiences through community engagement, and 6) guided learning with parents/caregivers through completion of a parent/caregiver-youth workbook. The allocation of learning opportunities to each study arm are detailed below and summarized in Table 1.

**Table 1:** Discover Comparative Effectiveness Trial: Intervention Components by Study Arm

	Components
Group A	• 6 after-school, large group sessions • *Ubongo Kids* videos on social emotional mindsets and skills
Group B	• 6 after-school sessions • *Ubongo Kids* videos on social emotional mindsets and skills • Mixed-gender, peer-guided group reflective discussions (4-5 participants)
Group C	• 18 after-school sessions (over 6 weeks) • *Ubongo Kids* videos on social emotional mindsets and skills • Mixed-gender experiential learning activities in small groups (4-5 participants) • Guided reflective discussions by trained facilitators to reinforce gender transformative content • Technology facilitated learning and mastery • Parent/caregiver-youth workbooks • Community Advisory Board and Community Event

#### Group A

Adolescents in Group A participated in six, two-hour learning sessions after school, (once a week for six weeks). In the sessions, participants watched *Ubongo Kids* videos on a projected screen in groups between 15-26. Each *Ubongo Kids* episode covered one of the social emotional mindsets and skills: teamwork, growth mindset, curiosity, persistence, purpose, and generosity, and an additional video on gender norms, beliefs and behaviors.

#### Group B

Adolescents in Group B participated in six, two-hour learning sessions after school (once a week for six weeks). In the sessions, participants watched *Ubongo Kids* videos projected on a screen *and* participated in peer-guided reflective discussions on the video content in small, mixed-gender groups of 4-5 participants. Themed discussion prompts corresponding to the respective social emotional mindset and skills content were provided to groups to guide reflections and facilitate discussion. While a trained facilitator was present, minimal facilitation was provided.

#### Group C

Adolescents in Group C participated in 18 two-hour sessions, delivered three times a week over six weeks. Participants viewed six *Ubongo Kids* videos projected on a screen in the classroom. After viewing the videos, participants were placed in small, mixed-gender groups of 4-5 participants. Each session included experiential learning activities completed in small mixed-gender groups and facilitated by a trained adult facilitator. Group C included reflective discussion guided by trained community facilitators to reinforce gender transformative learning. The Group C intervention also included a community and parent/caregiver component discussed below.

Community facilitators were trained with support from master trainers over eight days. During the training, six facilitation principles were adapted to facilitate discovery/experiential learning. The facilitation principles included: (1) scaffolded learning, (2) emphasis on learning over education, that is—facilitation emphasized the importance of the active process of experiential learning over teaching knowledge and skills, (3) withholding judgment and encouraging youth to take risks and learn from their mistakes and failures, (4) encouraging teamwork and positive group dynamics, (5) transformation of gender norms, beliefs and behaviors by challenging common practices such as boys taking credit for girls’ actions or ideas, and (6) encouraging a growth mindset and recognizing mastery of tasks achieved.

Experiential learning activities included use of tablets to scaffold exploration and mastery of social emotional mindsets and skills. Using technology, the intervention promoted exploratory learning through active practice by encouraging groups or pairs of youth to engage in games such as Tic-Tac-Toe and ping-pong, self-reflective activities such as mind-mapping, and creative expression including the design of fabric patterns using a drawing application. At the end of each session, students were guided by facilitators in reflective discussion that integrated gender transformative reflection. Reflective discussions utilized a variety of methods including drawing, free-writing, and pair sharing. The objective of the reflective discussions were to encourage adolescents to express their thoughts and feelings about the session’s content and activities, to recognize contributions of girls and boys to support more equitable gender beliefs and behaviors and to provide a space for celebrating achievements and mastery of skills.

Creative expression using tablets was designed to be culturally meaningful, and adolescents used the fabric design activity to work in small groups to create a *kanga*, a traditional Tanzanian fabric that is worn in a variety of ways or displayed in homes and communities. Kangas have important cultural significance in East Africa and often include a Swahili proverb meant to transfer wisdom from one generation to the next. *Discover* youth in Group C designed their own kanga, and a local printer printed the design on cloth to create the kanga. The kangas were given as gifts to participants, teachers and the community as a tangible embodiment of learning and achievements of adolescent participants.

The Group C intervention arm included engagement of parents/caregivers and the community. Parents/caregivers and community members were invited to attend biweekly meetings where *Discover* content was clarified and discussed to enhance parental/caregiver participation and to reinforce adolescent learning. Group C participants were provided with a parent/caregiver-youth workbook. The workbook included activities to complete with parents/caregivers and reflective discussion questions to reinforce learning content. Prior to the start of the intervention, *Discover* facilitated the creation of a Community Advisory Board (CAB) that included members of the local government, teachers and community members. At the end of the intervention, a community celebration event was held which brought together study participants, adolescents not participating in the intervention, parents/caregivers, teachers, education officials and other important community stakeholders. Adolescents presented what they had learned throughout the intervention, including showcasing their drawings and presenting their kangas to the community.

The three arm design of *Discover* was used to test the hypothesis that 1) social emotional learning is amplified through mixed-gender small group reflective discussion (Group B) in comparison to viewing content only (Group A), 2) experiential learning in small mixed-gender groups provides unique opportunities to reinforce gender transformative content and improve gender norms beliefs and behaviors (Group C), and, 3) parent/caregiver and community engagement can reinforce social emotional learning (Group C). The three-arm design allowed the study team to evaluate the relative impact of more resource intensive learning packages and to identify learning packages with the greatest impact on outcomes.

*Discover’s* theory of change posits that programs for very young adolescents before the onset of puberty (10-11 years of age) is a unique, culturally acceptable opportunity to provide experiential learning in mixed-gender groups that can be leveraged to promote gender equity and learning social emotional mindsets and skills that are foundational to supporting positive health trajectories. Furthermore, *Discover*’s theory of change posits that interventions targeting very young adolescents can protect against risks and vulnerabilities to health that intensify in mid to later adolescence including a broad array of outcomes such as mental health and well-being, education, economic and sexual and reproductive health outcomes.

### Setting

*Discover* was conducted in the Temeke Municipality in Dar es Salaam, Tanzania*.* The Temeke District is the largest of Dar es Salaam’s three districts and is unique because it encompasses both metropolitan urban and rural areas. There are 114 primary schools in Temeke providing education to 170, 477 primary school children [43]. The study was conducted between July and November of 2019. Four out of 114 schools in Temeke District were eligible to participate in the study. The eligibility criteria included, 1) School is a public school; 2) School’s population is representative of a typical school in peri-urban Tanzania in terms of demographics; 3) School has a large population to be able to obtain the desired sample of 10-11-year-olds; and 4) School is located in close proximity to where the majority of participants live. Schools were excluded from the study if they had participated in any behavioral change program within the last seven years (e.g. life skills training for students), or if they had an ongoing after-school program. All study activities, except for the parent/caregiver-youth workbook activities were carried out at the participants’ school location after school hours. The average classroom size across the four schools was 63 students.

#### Participant eligibility and recruitment

Participants were recruited from the four selected schools in the Temeke District of Dar es Salaam. Research assistants obtained written permission from parents/caregivers for their child to participate. To be sensitive to variations in literacy, and in accordance with local research practice, research assistants read parental/caregiver consent forms aloud and participants provided written signatures, or alternatively, a thumbprint to indicate consent. If parents/caregivers provided written consent, adolescent participants were read aloud the child assent form and provided verbal assent. Participants were eligible to participate in the study if they were 10-11 years old, were in grades 3, 4, or 5 and if parental/caregiver consent and adolescent assent were obtained. Participants were randomized to three arms of the study at the individual level. At each school, an event was held where eligible youth stood in lines outside their classrooms. A research assistant held a box with well-mixed pencil sharpeners of different colors inside. The box had a small hole large enough to fit a hand through but small enough that students could not see the pencil sharpeners. Each adolescent selected one sharpener at random. Adolescents were assigned to Groups A, B or C depending on whether they picked pink, yellow or blue pencil sharpeners respectively. Youth then matched their selected pencil sharpener with a research assistant holding the same color. Research assistants then registered students to one of the three groups.

#### Sample

**Fig. 1:**
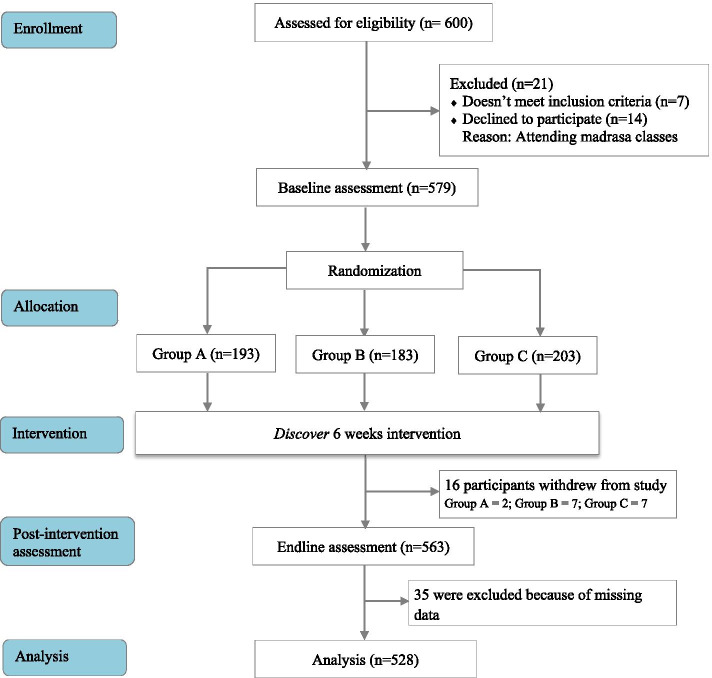
Discover Learning Study Flowchart

Power analysis determined that a minimum of 164 participants per study group would be sufficient to detect an effect size of 0.31 with a power of 80%, assuming a two-tailed significance level, α of 0.05. The effect size was calculated using the T statistic and non-centrality parameters for a paired two-sample t-test for the gender norms outcome, a primary outcome of interest for this study. Of the 600 youth that were contacted and invited to participate in the study, 14 declined and 7 did not meet the eligibility criteria. The remaining 579 adolescents completed the initial baseline assessment. 16 adolescents dropped out during the course of the intervention and did not complete the post-intervention assessment. Of the 579 who completed the baseline survey, 528 adolescents completed the endline survey that was used in this analysis. Figure 1 shows a detailed flow chart of recruitment, eligibility and randomization procedures.

### Data collection

Baseline data was collected from study participants in June-July 2019 at the study sites. Caregivers of participants consented to and supplied data on socio-economic status and demographic data during enrollment at baseline. The adolescent survey was administered to participants after informed consent was received from parents/guardians and verbal assent obtained from the participant. The adolescent survey included items from validated measures used in similar settings and were adapted after discussion with the Tanzanian research team. Pilot testing of study instruments were conducted with 60 adolescent participants in a primary school in Dar-es-Salaam with a similar socioeconomic profile as the study population. Translation and back translation of the questionnaire from English to Swahili was completed. Research assistants (RAs) were trained by lead Tanzanian research supervisors to conduct interviews using the study questionnaire. Training included a focus on research ethics, using tablets to collect data, discrete choice experiment training and referral to services for participants as appropriate. RAs pilot tested the questionnaire on a tablet and the survey instrument was refined to ensure comprehension of the questionnaire by very young adolescents. RAs administered the survey in Swahili, the local dialect. Responses were entered using a tablet and recorded in real time. At the end of the intervention, endline data was collected from study participants in September and October 2019. Privacy and confidentiality were observed and no information was shared outside the research team. All data was de-identified prior to data analysis. To compensate for time (45-60 min) participants received a small notebook and pen, approximately 2USD, after both baseline and endline surveys were completed. This compensation was an amount consistent with incentives for other studies in the area.

#### Study questionnaire

All participants completed a survey at baseline and after the intervention (VYA Questionnaire, Supplementary File 1). Measures related to social emotional skills and mindsets, and gender norms, beliefs and behaviors. Each measurement scale was selected based on previous research in low- and middle-income countries, and questions were adapted to the study context and age of participants.

### Survey measures

#### Growth mindset

Growth mindset was assessed using three adapted items from Dweck’s Growth Mindset measure (α= 0.96) [44]. Participants responded to statements reflecting growth mindset using a 4-point Likert scale (1 = *strongly disagree*; 4 *= strongly agree*). Score range is 3-9 with higher scores indicating greater growth mindset.

#### Purpose

The Dimensions of Identity Development Scale was adapted to assess the sense of purpose amongst adolescents in three domains (α= 0.71-0.90) [45]. The measure included a total of 6 questions. Respondents answered each item using a 4-point Likert scale ranging from 1, *strongly disagree* to 4, *strongly agree.* Score range is 6-24 with higher scores indicating greater purpose.

#### Goal orientation

The Goal Orientation and Learning Strategies Survey (GOALS-S) was adapted to measure four domains of goal orientation; Academic Goals, Social Goals, Cognitive Strategies and Meta-Cognitive Strategies (α= 0.90-0.98) [46]. Fourteen items were adapted for the study population and participants responded using a 4-point Likert scale was constructed for each item ranging from 1, (strongly disagree) to 4, (strongly agree). Score range is 14-24 with higher scores indicating greater goal orientation.

#### Curiosity

Ten items from the Gender-free Curiosity Inventory were adapted to measure curiosity in the sub-domains of; Exploration of the Complex, Conceptual Exploration, and, Perceptual Exploration (α= 0.84-0.87) [47]. Respondents answered each item using a 4-point Likert scale ranging from 1 (strongly disagree) to 4 (strongly agree) to capture participant’s recognition, pursuit and integration of new and challenging stimuli and experiences. Score range is 10-40 with higher scores indicating greater curiosity.

#### Persistence

Ten items from the Lufi and Cohen’s Persistence in Children measure were included (α= 0.66) [48]. Participants responded using a 4-point Likert scale (1=strongly disagree to 4 (strongly agree). Score range is 10-40 with higher scores indicating greater persistence.

#### Technology perceptions

20 items were selected from the original Media and Technology Usage and Attitudes Scale with 10 questions addressing technology usage (α=0.61) and 10 addressing technology perceptions and attitudes (α=0.97) [49]. Score range is 9-36 with higher scores indicating more positive technology perceptions.

#### Self-efficacy

All 24 questions from the original Self-Efficacy Questionnaire for Children (SEQ-C) scale were included in the adolescent survey (α= 0.85) [50]. Participants responded using a 5-point Likert scale (1- Not at all, 2-A little bit, 3-About average, 4-Well and 5-Very well). The SEQ-C measures emotional, academic and social self-efficacy. Score range is 24-120 with higher scores indicating greater self-efficacy.

#### Teamwork

13 items were included from the Teamwork and Collaboration Assessment for High School Students to capture three subdomains; cooperation, advocating/influence and negotiation among participants (α= 0.78-0.88) [51]. Score range is 13-52 with higher scores indicating more proficiency in teamwork.

#### Empathy

The Empathy Questionnaire for Children and Adolescents (EmQue-CA) was adapted to measure affective empathy, cognitive empathy, and intention to comfort (α= 0.70-0.74) [52]. Participants were asked to rate to what extent the statement was true for them on a 3-point scale: [1] not true, [2] sometimes, and [3] often true. Score range is 14-42 with higher scores indicating greater empathy.

#### Gender norms, beliefs and behaviors

13 items were adapted to measure gender norms, beliefs and behaviors among adolescents. Out of the 13 questions, 4 were adapted from the Gender Roles Equality and Transformations (GREAT) Project [53, 54], while 9 were developed and pretested based on context-specific gender norms in the areas of gender-roles, girls’ education and career prospective (α=0.81) [55]. Adapted items included questions such as, “Girls should have time to play with friends after school”, “Boys should have more free time overall than girls” and agree/disagree binary responses were recorded. Score range is 0-13 with higher scores indicating more positive, gender equitable beliefs and behaviors.

#### Generosity

The study adapted the Interpersonal Generosity Scale, a measure containing 10 items to capture six dimensions of interpersonal generosity; attention, compassion, open-handedness, self-extension, courage, and verbal expression using a standard Likert scale ranging from 1 (“strongly disagree”) to 4 (“strongly agree”) (α= 0.87) [56].

#### Bullying

The Global School-Based Students Health Survey (GSHS) was adapted to measure experiences of bullying among adolescents (α= 0.69-0.90) [57]. For this study, seven questions on bullying that were relevant to the context were selected. Students self-reported their responses to each question on a binary yes/no scale. Score range is 0-7 with higher scores indicating greater experiences of bullying.

### Additional measures

#### Discrete choice experiment

The survey contained 24 discrete choice experiment (DCE) questions to assess gender norms, beliefs and behaviors. DCE is a quantitative technique for eliciting individual preferences to inform policy, planning and resource allocation decisions [58]. Typically, in a DCE, study participants are repeatedly presented with scenarios on several attributes and asked to state their preference. In our approach, adolescents were presented with 3-5 scenarios for each of the social emotional mindsets and skills included in the content of the intervention and asked to decide whether the scenarios best described boys, girls, or both boys and girls. The items included scenarios related to curiosity, persistence, purpose, generosity, growth mindset/outlook on learning, teamwork and gender roles/responsibilities. Response options were presented on tablets as cartoon images of either boys, girls, or both girls and boys.

#### Psychological health

We assessed internalizing and externalizing behaviors using the African Youth Psychological Assessment (AYPA) Scale [59]. The survey adapted and refined 29 out of 41 culturally relevant questions to measure internalizing (α= 0.72) and externalizing symptoms (α= 0.88). The scale used a 4-point Likert scale of (1-Never, 2-Somewhat, 3-Often, 4-All the time). Score range was 0-19 for internalizing symptoms and 0-10 for externalizing symptoms with higher scores indicating more symptoms.

#### Parent/caregiver-youth workbook

For participants in Group C, an additional measure to assess engagement with the parent/caregiver-youth workbook was included.

#### Control variables

Control variables included age, gender, an adapted pubertal developmental scale [60], household size and socio-economic status (SES). SES was measured using the Tanzanian Simple Poverty Scorecard (α=0.57) [61]. The scale uses ten items including household composition, education, housing, ownership of durable assets, employment and agriculture to create a composite SES measure.

### Data analysis

Quantitative statistical data analysis was conducted using Stata Statistical Software: Release 15 by analysts at University of California Berkeley [62]. Data analysis used the per-protocol model to determine the effectiveness of the intervention on 1) Measures of social emotional mindsets and skills, and 2) gender norms, beliefs and behaviors. For primary analysis, data analysis included 528 participants. 51 participants were excluded from the analysis due to missing data and missing data was treated using the Pairwise Deletion method. This approach was selected because the missingness was at a person-level, and the response rate—defined as individuals who completed the survey was relatively high at 91% completion. Further, sensitivity analyses were conducted using complete cases to understand the degree of nonresponse bias likely present in the data. Descriptive statistics were calculated by intervention group at baseline and endline and the results were used to check for skewness and data non-normality. T-test, chi-square test and anova tests were used to assess and compare the impact of each intervention package on outcomes, controlling for age, gender and household poverty. Effect size estimates were calculated for each outcome measure using Cohen’s d, representing the standardized mean of intervention groups minus the standardized mean of the control group (Group A) and using pooled standard deviations for each study arm [63].

## Results

Five hundred and twenty-eight (528) participants completed both baseline and endline surveys and were included in the analysis. Survey data was collected from 179 participants in Group A, 158 participants in Group B, and 191 participants in Group C. 16 participants withdrew from the study before the intervention was completed. The most common reason for dropping out of the study was to attend Madrasa.

Demographic characteristics of the sample are summarized in Table 2. The final sample included 248 (47.0%) boys and 280 (53.0%) girls. The mean age of study participants was 10.5 (SD 0.5). The final sample included 261 (49.4%) 10-years old and 267 (40.6%) 11-years old. 153 (29.0%) of adolescents were from 3^rd^ grade, 241 (45.6%) were from 4^th^ grade and 134 (25.4%) were from 5^th^ grade. 345 (66.4%) of adolescents lived with both parents, compared to 173 (33.4%) who did not live with both parents. The average size of the sample’s household was 5.7 (SD 2.5). Study participants self-reported their general health on a scale of 1-4 (higher scores indicate better health), and participants reported a mean general health score of 1.94 (SD 0.98). Study participants scored on average 61.4 (SD 11.6) on the Tanzanian Poverty Scorecard (range 0-71; higher scores indicate less poverty). Adolescents were assessed for physical changes that occur during puberty using the Pubertal Development Scale (PDS), (range 1-3). As expected, differences in mean score for PDS were observed by gender. Girls reported a mean score of 0.82 (SD 0.85) and boys reported a mean score of 0.74 (SD 0.82). There were no significant differences across intervention arms for all socio-demographic measures. Age, grade, gender, living with both parents, household size, household poverty, general health and PDS covariates were assessed between intervention arms using Anova tests for group differences.

**Table 2:** Demographic Characteristics by Study Arm

	Group A (*N*=179)		Group B (*N*= 158)		Group C (*N*=191)		***Total*** (*N*=528)		*p*-value
	n	(%)	n	(%)	n	(%)	n	(%)	
**Gender**									0.330^Ω^
Boys	80	44.7	73	46.2	95	49.7	248	47.0	
Girls	99	55.3	85	53.8	96	50.3	280	53.0	
**Age**									0.250^Ω^
10	83	46.4	78	49.4	100	52.4	261	49.4	
11	96	53.4	80	50.6	91	47.6	267	50.6	
**Grade**									0.099^Ψ^
3^rd^	61	34.1	40	25.3	52	27.2	153	29.0	
4th	67	37.4	81	51.3	93	48.7	241	45.6	
5th	51	28.5	37	23.4	46	24.1	134	25.4	
**Live with Both Parents**									0.551^Ω^
No	60	34.9	53	33.5	60	31.9	173	33.4	
Yes	112	65.1	105	66.5	128	68.1	345	66.6	
**Total**	179	33.9	158	29.9	191	36.2	528	100.0	
	Mean (SD)		Mean (SD)		Mean (SD)		Mean (SD)		p-value
**Household profile**									
Household Size	5.6 (2.8)		5.8 (2.5)		5.8 (2.4)		5.7 (2.6)		0.800^ς^
Tanzanian Poverty Score (0-72)^1^	62.3 (11.6)		61.0 (11.8)		60.8 (11.5)		61.4 (11.6)		0.123^ς^
**Health**									
General Health Scale (1-4)	2.02 (0.99)		1.92 (0.99)		1.88 (0.96)		1.94 (0.98)		0.251^ς^
Pubertal Development Scale Girls (0-3)^2^	0.75 (0.85)		0.84 (0.90)		0.88 (0.80)		0.82 (0.85)		0.200^ς^
Pubertal Development Scale Boys (0-3)^2^	0.85 (0.92)		0.63 (0.79)		0.74 (0.76)		0.74 (0.82)		0.048^ς^

^1^Summative score of items 2-10 of the Tanzanian Poverty Scorecard [61]. Higher score=higher wealth

^2^Modified three question self-report PDS; Higher score=higher development

^Ω^Chi-square test p-value for differences in baseline characteristics by group assignment

^Ψ^ANOVA test p-value for differences in baseline characteristics by group assignment

^ς^T-test p-value for differences in baseline characteristics by group assignment

*p <0.05, **p < 0.01, ***p < 0.001; significant p-values are bolded

### Comparative Effectiveness Evaluation

Participant survey measures evaluated social emotional mindsets and skills at baseline and endline. Mean scores were reported for each outcome and treatment group. Overall, the results showed an increase in mean score from baseline to endline for most of the study outcomes. Participants in Group C showed greater change in mean scores for all outcomes compared to participants in Groups A and B. Tables 3, 4 and 5 show mean scores reported by participants before and after the intervention by assigned intervention group.

**Table 3:** Effect of Discover on Outcomes in Group A

	Baseline	Endline			
	Mean (SD)^1^	Mean (SD)^1^	β	p-value	Effect size^2^
**Adaptive Social Emotional Mindsets/Skills**					
Growth Mindset	20.3 (2.3)	20.4 (2.6)	0.06	0.407	0.02
Purpose	15.3 (2.8)	15.3 (2.6)	0.00	1.00	0.33
Goal Orientation	46.6 (4.9)	46.5 (5.2)	-0.11	0.422	0.03
Curiosity	31.0 (4.1)	31.2 (4.2)	0.23	0.302	0.30
Persistence	29.2 (3.5)	29.3 (3.7)	0.05	0.448	0.17
Self-Efficacy	92.6 (13.1)	97.5 (11.5)	4.93	0.001**	0.02
Technology Perceptions	20.5 (5.8)	23.0 (4.9)	2.50	<0.001***	0.14
Teamwork	39.9 (6.6)	41.3 (6.0)	1.33	0.023*	0.07
Empathy	17.4 (4.9)	17.8 (4.7)	0.74	0.036*	0.17
Gender Norms, Beliefs & Behaviors	6.4 (2.0)	7.3 (2.2)	0.87	0.000***	0.12
Generosity	31.9 (4.0)	31.6 (4.1)	-0.27	0.265	-0.22
Bullying	1.8 (1.8)	1.3 (1.4)	-0.52	0.001**	-0.09
**Psychological Assessment**					
Internalizing Symptoms	5.7 (7.4)	4.4 (6.5)	-1.33	<0.001***	-0.17
Externalizing Symptoms	3.2 (2.0)	3.0 (1.8)	-0.20	0.205	-0.10

^1^Mean of summative scores for measures

*p <0.05, **p<0.01, ***p<0.001; significant p-values are bolded

^2^Effect size estimate (Cohens’s d) was calculated as the difference between the standardized mean change for the treatment group in comparison to the control group (Group A)

**Table 4:** Effect of Discover on Outcomes in Group B

	Baseline	Endline			
	Mean (SD)^1^	Mean (SD)^1^	β	p-value	Effect size^2^
**Adaptive Social Emotional Mindsets/Skills**					
Growth Mindset	20.2 (2.4)	20.4 (2.5)	0.18	0.255	0.18
Purpose	15.5 (2.8)	16.2 (2.6)	0.69	0.011*	0.08
Goal Orientation	45.7 (4.8)	46.7 (5.0)	0.92	0.048*	0.08
Curiosity	30.8 (4.0)	32.4 (3.8)	1.63	0.000***	0.09
Persistence	29.6 (3.6)	29.9 (3.3)	0.31	0.212	0.12
Self-Efficacy	95.0 (11.1)	97.7 (11.5)	2.69	0.017*	0.10
Technology Perceptions	21.4 (5.1)	22.3 (4.8)	0.90	0.054	0.34
Teamwork	40.4 (6.3)	41.6 (5.7)	1.22	0.036*	0.24
Empathy	16.5 (5.1)	16.9 (4.7)	0.33	0.273	0.15
Gender Norms, Beliefs & Behaviors	6.6 (2.1)	7.6 (2.2)	0.91	0.000***	0.17
Generosity	31.6 (3.8)	32.5 (3.8)	0.92	0.017*	0.11
Bullying	1.4 (1.6)	1.1 (1.3)	-0.25	0.067	-0.08
**Psychological Assessment**					
Internalizing Symptoms	4.6 (5.6)	3.4 (5.1)	-1.22	0.021*	-0.04
Externalizing Symptoms	3.1 (2.1)	2.9 (1.5)	-0.20	0.164	-0.09

1Mean of summative scores for measures

*p <0.05, **p<0.01, ***p<0.001; significant p-values are bolded

^2^Effect size estimate (Cohens’s d) was calculated as the difference between the standardized mean change for the treatment group in comparison to the control group (Group A).

**Table 5:** Effect of Discover on Outcomes in Group C

	Baseline	Endline			
	Mean (SD)^1^	Mean (SD)^1^	β	*p*-value	Effect size^2^
**Adaptive Social Emotional Mindsets/Skills**					
Growth Mindset	19.9 (2.5)	20.9 (2.6)	1.00	0.000***	0.20
Purpose	15.5 (2.4)	16.4 (2.4)	0.90	0.000***	0.42
Goal Orientation	46.0 (4.6)	47.1 (5.3)	1.15	0.012*	0.11
Curiosity	30.9 (3.6)	32.0 (3.9)	1.10	0.002**	0.21
Persistence	29.0 (3.3)	30.3 (3.6)	1.41	<0.001***	0.28
Self-Efficacy	93.3 (12.3)	98.7 (9.7)	5.38	<0.001***	0.11
Technology Perceptions	20.6 (5.3)	23.9 (4.5)	3.25	<0.001***	0.19
Teamwork	39.2 (6.4)	43.0 (5.6)	3.76	<0.001***	0.30
Empathy	16.6 (4.8)	17.7 (4.9)	1.04	0.020*	0.01
Gender Norms, Beliefs & Behaviors	6.1 (2.0)	7.9 (2.3)	1.79	<0.001***	0.28
Generosity	31.3 (3.5)	32.9 (3.8)	1.61	<0.001***	0.32
Bullying	1.8 (1.8)	1.2 (1.5)	-0.52	0.001**	-0.02
**Psychological Assessment**					
Internalizing Symptoms	5.2 (6.5)	3.2 (4.0)	-2.01	0.000***	-0.22
Externalizing Symptoms	3.2 (1.9)	2.7 (1.4)	-0.45	0.004**	-0.18

^1^Mean of summative scores for measures

*p <0.05, **p<0.01, ***p<0.001; significant p-values are bolded

^2^Effect size estimate (Cohens’s d) was calculated as the difference between the standardized mean change for the treatment group in comparison to the control group (Group A)

For most outcomes, there were statistically significant improvements in social emotional mindsets and skills post-intervention. Measures that were reverse-coded (internalizing symptoms, externalizing symptoms and bullying) indicated a decrease in mean internalizing symptom in all three groups and a decrease in mean externalizing symptoms in Group C only. Participants in Group C showed larger improvements in most study outcomes compared to those in Groups A and B. These findings support our hypothesis that participants in Group C would demonstrate greater change in social emotional mindsets and skills in comparison to those in Groups A and B. Changes in mean score for Goal Orientation and Generosity measures did not reach significance among participants in Group A, but showed statistically significant improvements in Groups B and C.

Table 6 shows the results from a baseline-adjusted analysis, controlled for age and household poverty. Household poverty and age were fitted in the regression model as covariates. Estimated effects of the intervention were consistent in direction but attenuated compared to estimates from an unadjusted model. Group C, which received the highest level of intervention showed the largest effect sizes. Group B showed smaller effect sizes compared to Group C, but larger than Group A. In this model, Group A was treated as the control group because participants received the lowest level of intervention (watching video episodes), and the video episodes are widely available throughout Tanzania.

**Table 6:** Change in adjusted mean score by study arm

	Intervention groups				Control group	
	Group C		Group B		Group A	
	Mean ∆	Effect size	Mean ∆	Effect size	Mean ∆	Effect size
Growth Mindset	0.94	1.09	-0.02	0.15	0.34	0.93
Purpose	0.91	3.33	0.69	2.55	-0.06	0.61
Goal Orientation	1.20	0.68	0.81	0.16	-0.19	0.52
Curiosity	1.12	1.85	1.67	2.64	0.17	0.86
Persistence	1.73	1.46	2.59	0.81	1.91	0.62
Self-Efficacy	5.27	0.45	2.72	0.15	4.55	0.33
Technology Perceptions	3.19	2.25	0.98	2.43	2.54	4.77
Teamwork	3.80	1.39	1.08	0.22	1.31	1.21
Empathy	1.15	0.08	0.47	0.70	0.59	0.63
Gender Norms, Beliefs and Behaviors	1.80	1.03	0.88	0.40	0.85	0.62
Generosity	1.64	2.04	0.94	1.34	0.28	0.64
Bullying	-0.52	-0.07	-0.24	-0.52	-0.52	-0.45
Internalizing symptoms	-2.06	-1.90	-1.33	-1.75	-1.47	-0.26
Externalizing symptoms	-0.47	-1.57	-0.24	-0.88	-0.15	-0.66

a) Mean ∆ is the change in adjusted mean scored(posttest score – pretest score).

b) Effect size is calculated using Cohen’s d.

### Gender norms, beliefs and behaviors

**Fig. 2:**
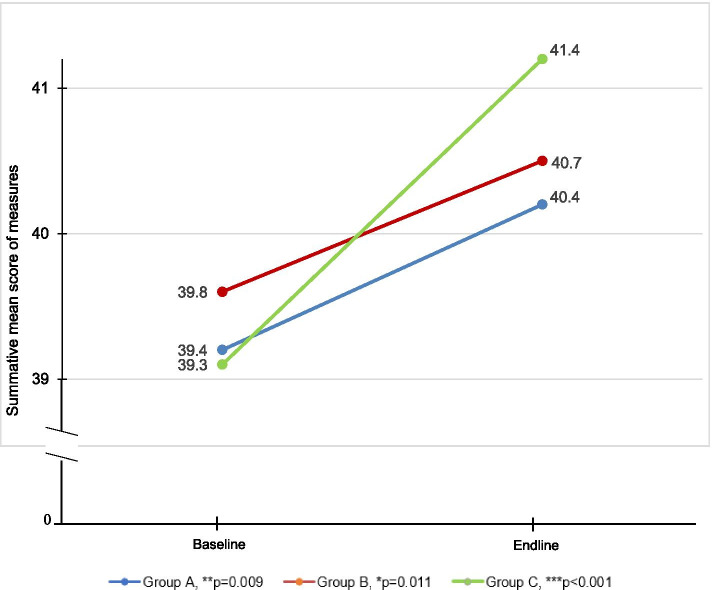
Discrete Choice Experiment Results: Change in More Equitable Gender Norms, Beliefs and Behaviors by Study Arm

Results from the discrete choice experiment revealed a significant change in mean score from baseline to endline in rating of the scenarios (as best describing boys, girls or both boys and girls) for all of the outcomes of the social emotional skills and mindsets. Summative scores for each participant were calculated to reflect a total range of 0 to 52. For all 26 questions, response options included *boys only* or *girls only (score=0)*, or, both *boys and girls* (score=1). A higher score reflects *positive* gender norms, beliefs and behaviors. In Group A, the average score was 15.36 (SD 7.40) at baseline and 19.63 (SD 7.14) post-intervention. Participants in Group B showed a slightly larger change from 14.65 (SD 8.04) at baseline to 19.14 (SD 7.87) at endline. Group C showed the most significant improvement from 14.04 (SD 7.49) at baseline to 21.13 (SD 6.73) post-intervention. These results support the hypothesis that *Discover* would have a positive impact on participant’s gender norms, beliefs and behaviors. A summary of results from the discrete choice experiment is shown in Figure 2.

## Discussion

Findings from *Discover* compare the effectiveness of three different intervention approaches designed for very young adolescents to increase social emotional mindsets and skills that a) support transformation in gender norms, beliefs and behaviors and b) support acquisition of social emotional mindsets and skills that promote adaptive development. Throughout the research design process, findings from adolescent developmental science were leveraged to design an intervention effective for very young adolescents. This period of brain development represents a unique opportunity for learning and experiences that actively shape developing neural networks involved in the processing of emotions, risks, rewards and social relationships that can have enduring impacts on health trajectories [11, 64, 65].

The transition from childhood into adolescence presents a unique opportunity to transform gender norms that are learned and shaped by the different lived experiences of girls and boys during a formative period of development. A key aspect of brain plasticity involves sensitive periods of learning—when learning--and brain development *interact* with experiences in formative ways. The onset of puberty signals a sensitive period of social and emotional learning; it is also a time when social experiences, roles, and responsibilities often divide across gender. Gender norms common among parents/caregivers and the community often amplify the separation of genders as girls and boys approach sexual maturity. Therefore, targeting adolescents near the onset of puberty is a particularly valuable window of time to design interventions that combine learning social emotional mindsets and skills and gender transformative content through experiential learning in mixed-gender group. Creating these learning opportunities near the onset of adolescence, may have enduring impacts on social, emotional mindsets and skills that support healthy identity development. We believe this approach can empower girls to realize their full potential and simultaneously protect against health risks that disproportionally affect girls. For example, Tanzania has one of the highest child marriage rates in the world and it is estimated that 20-40% of girls are married before their 18^th^ birthday [28, 66]. This is particularly problematic because women who bear children between 15-19 years old have higher mortality rates in comparison to other age groups [67]. Furthermore, sexual coercion is an enduring problem in Tanzania. For example, in the Mwanza Region, 30% of adolescent girls reported being forced in to their first sexual experience [68]. Mixed-gender experiential learning that incorporates gender transformative content can shape gender norms, beliefs and behaviors of both girls and boys and improve educational and economic outcomes.

**Fig. 3:**
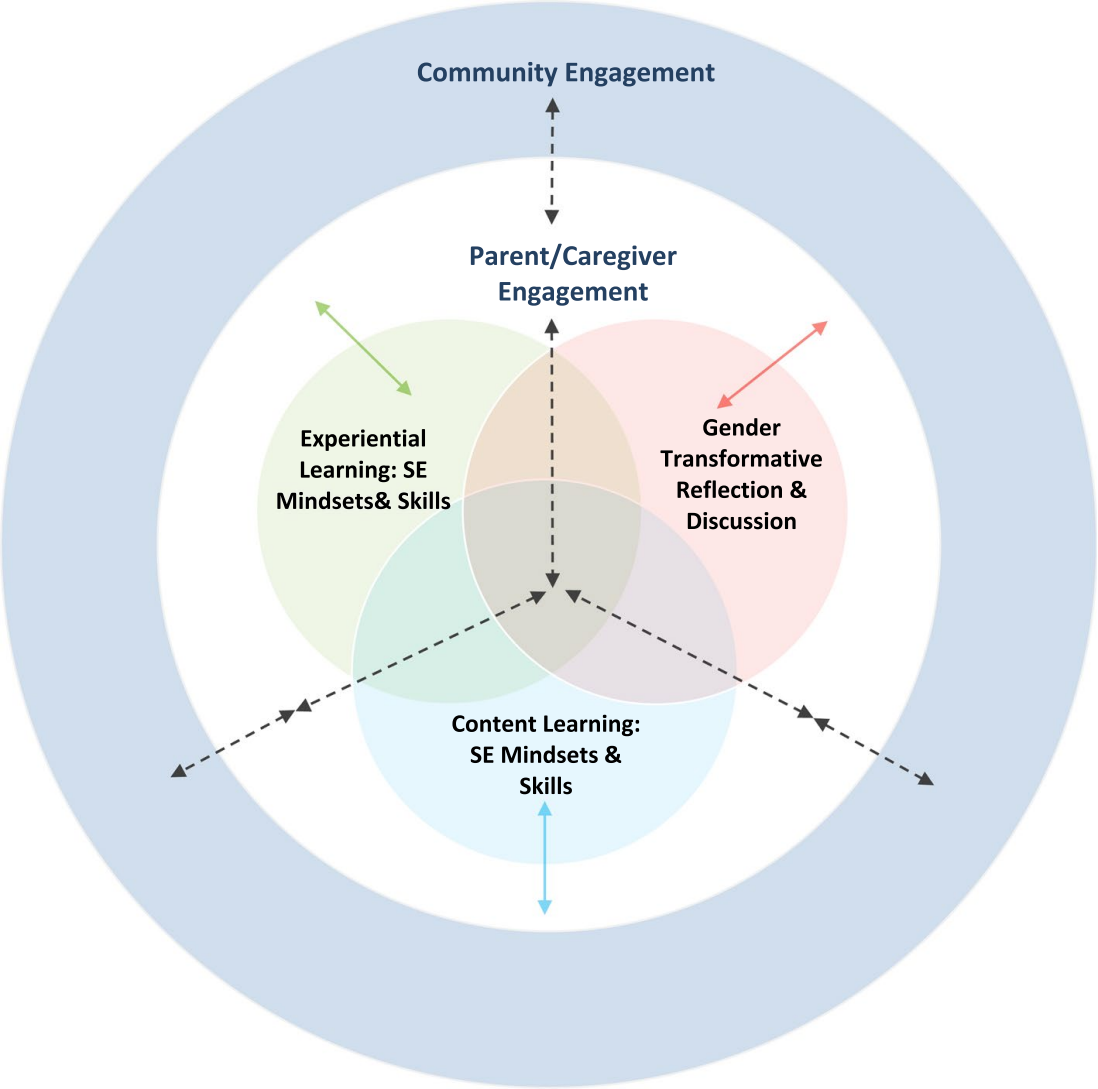
A multi-level implementation model of social emotional learning for very young adolescents

The findings from this comparative effectiveness trial provide insight into the added value of additional learning opportunities for reflection and discussion, experiential learning in mixed-gender groups, and involvement of parents/caregivers and the community in intervention approaches (Table 6 and Figure 3). Results from this study (in a low resource setting) highlight how approaches to learning impact the magnitude of effect on social emotional mindsets and skills and gender equity outcomes. Group A received content related to social emotional mindsets and skills only; Group B included the additional component of reflection and discussion in mixed gender groups; and Group C received the additional component of opportunities for experiential learning in mixed gender peer groups, with parents/caregivers and with the community. While all groups demonstrated improvement on key outcome measures, Group B demonstrated improvement across more measures and a greater overall improvement compared to Group A. These results are consistent with previous research demonstrating the critical importance of reflection after learning to maximize positive effects [69].

Providing opportunities to actively practice learning (Group C) amplified the impact on measures of social emotional mindsets and skills and gender norms, beliefs and behaviors (compared to Groups A and B). Experiential learning in mixed-gender groups, provides adolescents with opportunities to practice learning in a social context, building positive relationships with peers and to experience the equal and important contributions of both boys and girls. Facilitation by young adults emphasized prosocial behaviors and guided reflective activities the emphasized gender equity. Additional experiential learning and reflective discussions with parents/caregivers allowed reinforcement of learning within the home.

We hypothesize that part of the reason Group C had greater improvements on outcomes in comparison to Group A and B, is because experiential learning is particularly important for learning about self and others during adolescence [13]. Experiential learning is enhanced when adolescents are motivated to demonstrate their natural curiosity, engage in positive risk taking, embrace a growth mindset that encourages persistence and experience the thrill of mastering a new skill in a social context that is especially salient during adolescence. For example, recent research recognizes a natural increase in the desire to contribute meaningfully during adolescence; to “matter” [70]. Research from developmental science has found that adolescents are particularly sensitive to admiration by peers and adolescents are motivated to by opportunities to gain social status in ways that enhance the salience and impact of those social learning experiences [64, 71, 72].

### Amplifying learning opportunities during the transition from childhood to adolescence

The conceptual framework for the design of this study swas based on findings from developmental science demonstrating the importance of the developmental period near the onset of puberty. More specifically, we hypothesized that targeting this transition could leverage windows of opportunity for particular types of learning. *Discover* provided opportunities to increase social emotional knowledge, skills, and mindsets, but also provided experiential learning opportunities for positive risk taking in peer groups (Group C). Technology was incorporated in the Group C design by using tablets to provide a novel experience of discovery learning. Technology also leverages adolescents’ increased tendency to explore, take risks and master new skills. For example, playing games that required multiple attempts and different paths to success allowed adolescents to experience failure and success in peer groups. These activities also supported adolescents’ natural tendency to seek mastery, especially in a context where activities required collective contribution in small groups to solve problems and where risk-taking, innovation and mastery of skills were rewarded by peers. Developmental science has provided evidence that the dopaminergic reward system is particularly sensitive to exploratory learning experiences and is further enhanced by the presence of peer and social rewards [73]. Providing scaffolded, facilitated discussion that addressed the equal contribution of girls and boys in *Discover* experiential learning activities was an additional opportunity to reinforce gender transformative content and recognize achievements of girls in a mixed-gender peer context. In addition, each session of *Discover* ended with a gratitude exercise where participants reflected on and expressed gratitude for their peers, further leveraging adolescent’s natural desire for peer admiration and social reward. Intentionally designing the intervention to leverage adolescent sensitivity to social reward likely contributed to the greater effect size of the intervention in Group C in comparison to Groups A and B.

Through inclusion of parents/caregivers and the community in Group C, our results support recommendations for inclusion of parents/caregivers and the community. Reinforcement of learning in different social and family contexts can complement experiential learning in peer groups. Bronfenbrenner’s ecological model of human development recognizes the importance of factors at the individual, microsystem (family and peer relationships), mesosystem and macro level in child and adolescent development outcomes [74]. While research in high-resource contexts provides evidence for the importance of parental engagement for positive adolescent development, less research has focused on the importance of parental engagement in low-resource contexts [75]. Particularly in low-resource contexts in East Africa, community members serve an integral role in child and adolescent development [76, 77]. Engagement with parents/caregivers and community members in the design of *Discover* and involvement in the intervention resulted in participatory ownership of the intervention and opportunities to share the experience of learning with adolescents. The final community celebration event allowed Group C to present a culturally grounded, highly meaningful artifact, the kanga, to their community. Not only did this experience take advantage of adolescents’ sensitivity to reward and admiration by peers and adults, but it simultaneously provided the opportunity to demonstrate their value and contribution to their community – to *matter* – in peer groups, in their household and in the community.

### Limitations

Randomization of adolescents was completed at the individual level. Findings from the intervention apply to the individual level but cannot be extrapolated to the community level. Despite efforts to ensure intervention groups received the package of components pertinent to that group, some adolescents may have discussed their group’s activities with friends and siblings and therefore groups may have been aware of intervention components received by other groups. Longitudinal data is required to assess whether changes in social emotional mindsets and skills and/or gender norms, beliefs and behaviors are sustained over time. It is possible that “booster” interventions at future timepoints could help to sustain the gains in study outcomes. Employing the Pairwise Deletion method to treat missing data could have introduced bias and impacted the effect size of treatment group outcomes for this study. To control for potential bias, we conducted sensitivity analyses on complete-case data and found no significant differences in the results. Intervention components were tailored to the Tanzanian context and may not be generalizable to other contexts without similar adaptation of measures, content and program delivery.

## Conclusion

Findings from this study indicate that interventions with very young adolescents can benefit from including experiential learning opportunities in small-mixed gender groups. This study demonstrates that integration of gender transformative content with social emotional learning content can promote more gender equitable norms, beliefs and behaviors. Leveraging findings from developmental science can increase the effectiveness of interventions designed for very young adolescents. Targeting programs for very young adolescents can support positive trajectories for a range of health outcome. Future investments in adolescent programing should consider designing interventions for specific periods of adolescent development and explore how sequencing of interventions across adolescent development can amplify and sustain the effect of interventions on positive health trajectories.

## Supplementary Information

**Additional file 1.**

## Data Availability

The datasets generated and/or analyzed during the current study are not publicly available due to the sensitive age of the study participants (10-11-year-old) at baseline but are available from the corresponding author on reasonable request. The author will vet requests to be certain that appropriate IRB approvals and data safety guidelines are in place before distribution.

## Abbreviations

CAB: Community Advisory Board
DCE: Discrete Choice Experiment
HPON: Health for a Prosperous Nation
ICT: Information and Communication Technology
PDS: Pubertal Development Scale
Ras: Research assistants
RCT: Randomized controlled Trial
SES: Socio-economic Status
SPIRIT: Standard Protocol Items: Recommendations for Interventional Trials
UC Berkeley: University of California Berkeley
VYAs: Very Young Adolescents

## References

[1] Patton GC, Sawyer SM, Santelli JS, Ross DA, Afifi R, Allen NB (2016). Our future: a Lancet commission on adolescent health and wellbeing. Lancet (London, England)..

[2] Unicef. The State of the World's Children (2019). Children, Food and Nutrition - Growing Well in a Changing World. UN, New York.

[3] National Academies of Sciences E, Division HaM, Education DoBaSSa, Board on Children Y, Applications CotNaS-bSoADaI, Backes EP, et al. Introduction: National Academies Press (US); 2019 2019/05/16/.

[4] Shah R, Hagell A, Cheung R. International comparisons of health and wellbeing in adolescence and early adulthood2019 2019/02/24/.

[5] Bashir S. Changing the Trajectory: Education and Training for Youth in Democratic Republic of Congo: World Bank Publications; 2009 2009/06/25/. 98 p.

[6] Yeager DS (2017). Social and Emotional Learning Programs for Adolescents. The Future of Children..

[7] Blakemore S-J, Mills KL (2014). Is Adolescence a Sensitive Period for Sociocultural Processing?. Annual Review of Psychology..

[8] Dahl RE, Allen NB, Wilbrecht L, Suleiman AB (2018). Importance of investing in adolescence from a developmental science perspective. Nature..

[9] Piekarski DJ, Johnson CM, Boivin JR, Thomas AW, Lin WC, Delevich K, et al. Does puberty mark a transition in sensitive periods for plasticity in the associative neocortex? Brain research. 2017;1654(Pt B):123-44. 10.1016/j.brainres.2016.08.042 PMC5283387 27590721

[10] Peeters M, Oldehinkel A, Veenstra R, Vollebergh W (2019). Unique developmental trajectories of risk behaviors in adolescence and associated outcomes in young adulthood. PLOS ONE..

[11] Crone EA, Dahl RE (2012). Understanding adolescence as a period of social–affective engagement and goal flexibility. Nature Reviews Neuroscience..

[12] Yeager DS, Hanselman P, Walton GM, Murray JS, Crosnoe R, Muller C (2019). A national experiment reveals where a growth mindset improves achievement. Nature..

[13] Crone EA, Fuligni AJ (2020). Self and Others in Adolescence. Annual Review of Psychology..

[14] Dweck CS, Yeager DS (2019). Mindsets: A View From Two Eras. Perspectives on Psychological Science..

[15] Viner RM, Allen NB, Patton GC. Puberty, Developmental Processes, and Health Interventions. In: Bundy DAP, Silva Nd, Horton S, Jamison DT, Patton GC, editors. Child and Adolescent Health and Development. 3rd ed. Washington (DC): The International Bank for Reconstruction and Development / The World Bank; 2017. 30212144

[16] Kågesten AE, Pinandari AW, Page A, Wilopo SA, van Reeuwijk M (2021). Sexual wellbeing in early adolescence: a cross-sectional assessment among girls and boys in urban Indonesia. Reprod Health..

[17] Cherewick M, Lebu S, Su C, Richards L, Njau PF, Dahl RE (2021). Adolescent, caregiver and community experiences with a gender transformative, social emotional learning intervention. International Journal for Equity in Health..

[18] Stark L, Asghar K, Seff I, Yu G, Tesfay Gessesse T, Ward L (2018). Preventing violence against refugee adolescent girls: findings from a cluster randomised controlled trial in Ethiopia. BMJ Glob Health..

[19] Langford R, Bonell CP, Jones HE, Pouliou T, Murphy SM, Waters E (2014). The WHO Health Promoting School framework for improving the health and well-being of students and their academic achievement. Cochrane Database Syst Rev..

[20] Niemiec RM, Pearce R (2020). The Practice of Character Strengths: Unifying Definitions, Principles, and Exploration of What's Soaring, Emerging, and Ripe With Potential in Science and in Practice. Front Psychol..

[21] Moreau C, Li M, De Meyer S, Vu Manh L, Guiella G, Acharya R, et al. Measuring gender norms about relationships in early adolescence: Results from the global early adolescent study. SSM Popul Health. 2019;7:014-. 10.1016/j.ssmph.2018.10.014 PMC6293033 30581959

[22] Blum RW (2020). Gender Norm Transformative Programing: Where Are We Now? Where Do We Need to Be?. Journal of Adolescent Health..

[23] Gupta AK, Santhya KG (2020). Promoting Gender Egalitarian Norms and Practices Among Boys in Rural India: The Relative Effect of Intervening in Early and Late Adolescence. Journal of Adolescent Health..

[24] Sommer M (2011). An Overlooked Priority: Puberty in Sub-Saharan Africa. American Journal of Public Health..

[25] Mwale M, Muula AS (2017). Systematic review: a review of adolescent behavior change interventions [BCI] and their effectiveness in HIV and AIDS prevention in sub-Saharan Africa. BMC Public Health..

[26] Aslam RW, Hendry M, Booth A, Carter B, Charles JM, Craine N, et al. Intervention Now to Eliminate Repeat Unintended Pregnancy in Teenagers (INTERUPT): a systematic review of intervention effectiveness and cost-effectiveness, and qualitative and realist synthesis of implementation factors and user engagement. BMC Medicine. 2017;15. 10.1186/s12916-017-0904-7 PMC5557469 28806964

[27] Kabiru CW, Izugbara CO, Beguy D (2013). The health and wellbeing of young people in sub-Saharan Africa: an under-researched area?. BMC International Health and Human Rights..

[28] MoHCDGEC. Tanzania Demographic and Health Survey and Malaria Indicator Survey 2015-2016. Dar es Salaam, Tanzania: Ministry of Health, Community Development Gender Elderly and Children (MoHCDGEC) Tanzania Mainland Ministry of Health - MoH. Zanzibar National Bureau of Statistics, N.B.S. Tanzania Office of Chief Government Statistician, OCGS Zanzibar ICF; 2016 2016.

[29] UNDP. Tanzania Human Development Report 2017 - Social Policy in the Context of Economic Transformation. United Nations Development Programme. Tanzania Office, Economic and Social Research Foundation; 2018 2018. Report No.: 978-9976-5231-8-8.

[30] Ferrone L, de Milliano M (2018). Multidimensional Child Poverty in three Countries in Sub-Saharan Africa. Child indicators research..

[31] UNICEF. Global Initiative on Out-of_School Children. Tanzania Country Report. Dar es salaam: Ministry of Education Sciience and Technoloogy; 2018 2018.

[32] HRW. “I Had a Dream to Finish School”. Barriers to Secondary Education in Tanzania. 2017 2017/02/14/T02:01:00-0500.

[33] Mbelle A. School enrolment, performance and access to education in Tanzania. Dar es Salaam, Tanzania :: Mkuki na Nyota Publishers; 2003 c2003.

[34] Al-Samarrai S, Tamagnan ME, editors. Gender Equity and Fee-Free Basic Education in Tanzania Summary2019 2019.

[35] Lowe MC, Danny G. SADC Gender Protocol 2018 Barometer: Gender Links; 2018 2018/08/20/. 345 p.

[36] UNFPA. Fact Sheet: Teenage Pregnancy. Tanzania: United Nations Population Fund; 2018 2018.

[37] Blum RW, Mmari K, Moreau C (2017). It Begins at 10: How Gender Expectations Shape Early Adolescence Around the World. Journal of Adolescent Health..

[38] Schlecht J, Lee C, Kerner B, Greeley M, Robinson C (2017). Prioritizing programming to address the needs and risks of very young adolescents: a summary of findings across three humanitarian settings. Conflict and Health..

[39] Mtebe J, Raphael C. A Critical Review of eLearning Research Trends in Tanzania. Journal of Learning for Development - JL4D. 2018;5:163-78.

[40] Burns M, Santally M, Rajabalee Y, Halkhoree R, Sungkur R. Background Paper Information and Communications Technologies in Secondary Education in Sub-Saharan Africa Policies, Practices, Trends, and Recommendations2019.

[41] Claudius Komba S, Dave DN (2016). The use of computers by primary school pupils in Morogoro, Tanzania. International Journal of Research Studies in Educational Technology..

[42] Cherewick M, Lebu S, Su C, Dahl RE (2020). An Intervention to Enhance Social, Emotional, and Identity Learning for Very Young Adolescents and Support Gender Equity: Protocol for a Pragmatic Randomized Controlled Trial. JMIR Res Protoc..

[43] Rwechungura JK. An exploratory study of the factors contributing to school dropout among girls in Temeke district of Dar es Salaam, Tanzania. 2014.

[44] Dweck CS, C-y C, Y-y H (1995). Implicit Theories and Their Role in Judgments and Reactions: A Word From Two Perspectives. Psychological Inquiry..

[45] Mastrotheodoros S, Motti-Stefanidi F (2017). Dimensions of Identity Development Scale (DIDS): A test of longitudinal measurement invariance in Greek adolescents. European Journal of Developmental Psychology..

[46] Dowson M, McInerney DM (2004). The Development and Validation of the Goal Orientation and Learning Strategies Survey (Goals-S). Educational and Psychological Measurement..

[47] Kashdan T, Stiksma M, Disabato D, McKnight P, Bekier J, Kaji J, et al. The Five-Dimensional Curiosity Scale: Capturing the bandwidth of curiosity and identifying four unique subgroups of curious people. Journal of Research in Personality. 2017;73.

[48] Lufi D, Cohen A. A Scale for Measuring Persistence in Children. Journal of Personality Assessment. 1987;51(2):178-=85. 10.1207/s15327752jpa5102_2 16372846

[49] Rosen LD, Whaling K, Carrier LM, Cheever NA, Rokkum J (2013). The Media and Technology Usage and Attitudes Scale: An empirical investigation. Comput Human Behav..

[50] Minter A, Pritzker S (2017). Measuring Adolescent Social and Academic Self-Efficacy: Cross-Ethnic Validity of the SEQ-C. Research on Social Work Practice..

[51] Zhuang X, MacCann C, Wang L, Liu L, Roberts RD. Development and Validity Evidence Supporting a Teamwork and Collaboration Assessment for High School Students. ETS Research Report Series. 2008;2008(2):i-51.

[52] Overgaauw S, Rieffe C, Broekhof E, Crone EA, Güroğlu B. Assessing Empathy across Childhood and Adolescence: Validation of the Empathy Questionnaire for Children and Adolescents (EmQue-CA). Front Psychol. 2017;8. 10.3389/fpsyg.2017.00870 PMC5447078 28611713

[53] Scales PC, Shramko M, Ashburn K (2016). Developmental Assets and Sexual and Reproductive Health among 10- To 14-Year-Olds In Northern Uganda. International Journal of Child, Youth and Family Studies..

[54] Pulerwitz J, Barker G. Measuring Attitudes toward Gender Norms among Young Men in Brazil: Development and Psychometric Evaluation of the GEM Scale. Men and Masculinities. 2007.

[55] Lundgren R, Beckman M, Chaurasiya SP, Subhedi B, Kerner B (2013). Whose turn to do the dishes? Transforming gender attitudes and behaviours among very young adolescents in Nepal. Gender & Development..

[56] Smith C, Hill JP, editors. Toward the Measurement of Interpersonal Generosity (IG): An IG Scale Conceptualized, Tested, and Validated2009 2009.

[57] Brown D, Riley L, Kann L (2008). Bullying among youth from eight African countries and associations with adverse health behaviors. Pediatric Health..

[58] Mangham LJ, Hanson K, McPake B (2009). How to do (or not to do) ... Designing a discrete choice experiment for application in a low-income country. Health Policy Plan..

[59] Betancourt TS, Yang F, Bolton P, Normand SL (2014). Developing an African youth psychosocial assessment: an application of item response theory. Int J Methods Psychiatr Res..

[60] Petersen AC, Crockett L, Richards M, Boxer A (1988). A self-report measure of pubertal status: Reliability, validity, and initial norms. Journal of Youth and Adolescence..

[61] Schreiner M (2006). A Simple Poverty Scorecard for Haiti.

[62] StataCorp. Stata Statistical Software. 15 ed. College Station, TX, USA: StataCorp LLC; 2017.

[63] Morris SB (2008). Estimating Effect Sizes From Pretest-Posttest-Control Group Designs. Organizational Research Methods..

[64] Braams BR, van Duijvenvoorde ACK, Peper JS, Crone EA (2015). Longitudinal Changes in Adolescent Risk-Taking: A Comprehensive Study of Neural Responses to Rewards, Pubertal Development, and Risk-Taking Behavior. J Neurosci..

[65] Spielberg JM, Olino TM, Forbes EE, Dahl RE (2014). Exciting fear in adolescence: does pubertal development alter threat processing?. Developmental Cognitive Neuroscience..

[66] Girls Not Brides. It Takes a Movement: Reflecting on Five Years of Progress Towards Ending Child Marriage. London: Girls Not Brides; 2016 2016.

[67] Sathiya Susuman A, Hamisi HF (2012). Under-5 mortality in Tanzania: a demographic scenario. Iran J Public Health..

[68] Wight D, Plummer ML, Mshana G, Wamoyi J, Shigongo ZS, Ross DA (2006). Contradictory sexual norms and expectations for young people in rural Northern Tanzania. Soc Sci Med..

[69] van Goethem A, van Hoof A (2014). Orobio de Castro B, Van Aken M, Hart D. The role of reflection in the effects of community service on adolescent development: a meta-analysis. Child Dev..

[70] Fuligni AJ. The Need to Contribute During Adolescence. Perspectives on Psychological Science. 2018. 10.1177/1745691618805437 PMC6497551 30562473

[71] Casey BJ, Galván A, Somerville LH (2016). Beyond simple models of adolescence to an integrated circuit-based account: A commentary. Developmental Cognitive Neuroscience..

[72] Shulman EP, Smith AR, Silva K, Icenogle G, Duell N, Chein J (2016). The dual systems model: Review, reappraisal, and reaffirmation. Developmental Cognitive Neuroscience..

[73] van Duijvenvoorde ACK, Peters S, Braams BR, Crone EA (2016). What motivates adolescents? Neural responses to rewards and their influence on adolescents' risk taking, learning, and cognitive control. Neurosci Biobehav Rev..

[74] Bronfenbrenner U, Morris PA. The Bioecological Model of Human Development. In: Damon W, Lerner RM, editors. Handbook of Child Psychology. Hoboken, NJ, USA: John Wiley & Sons, Inc.; 2007. p. chpsy0114.

[75] Fulu E, Miedema S, Roselli T, McCook S, Chan KL, Haardörfer R (2017). Pathways between childhood trauma, intimate partner violence, and harsh parenting: findings from the UN Multi-country Study on Men and Violence in Asia and the Pacific. The Lancet Global Health..

[76] Somefun OD, Odimegwu C (2018). The protective role of family structure for adolescent development in sub-Saharan Africa. PLOS ONE..

[77] Atilola O. Where Lies the Risk? An Ecological Approach to Understanding Child Mental Health Risk and Vulnerabilities in Sub-Saharan Africa. Psychiatry Journal. 2014. 10.1155/2014/698348 PMC4009193 24834431

