# Supplementary material for: Promoting gender equity in very young adolescents: targeting a window of opportunity for social emotional learning and identity development
Source: BMC Public Health. 2021 Dec 19;21:2299. doi: 10.1186/s12889-021-12278-3 (PMC8684613; doi:10.1186/s12889-021-12278-3)
Supplement: Supplementary file 1 — Additional file 1. [file 12889_2021_12278_MOESM1_ESM.doc]

**Discover Learning**

**Very Young Adolescent Questionnaire**

Notes:

- 996 = Refuse to answer or no response, is available for every question.

| Youth ID Number: |  |
| --- | --- |
| Interviewer Name: |  |
| Interview Date: | (day/month/year) |
| Interview Start Time: |  |
| Interview End Time: |  |

**I. Sociodemographic**

SD1. How old are you?

NUMERICAL (years)

SD2. Are you a…?

0 = Boy

1 = Girl

SD3. What grade are you in?

0 = 3rd

1 = 4th

2 = 5^th^

**II. Health**

H1. In general, how is your health?

0 = Poor

1 = Fair

2 = Good

3 = Excellent

**For girls only:**

H2. Have you recently started to grow taller quickly?

0 = No

1 = Yes

H3. Have you recently started changing from looking like a girl to looking like a woman?

0 = No

1 = Yes

H4. Have you started to have periods?

0 = No

1 = Yes

H5. If Yes to (got period)] How old were you when you first got your period?

NUMERICAL [limit 7‐12 years old]

**For boys only:**

H6. Have you recently started changing from looking like a boy to looking more like a man?

0 = No

1 = Yes

H7. Has your voice become deeper recently?

0 = No

1 = Yes

H8. [If Yes to (speak in a deeper voice)] How old were you when your voice started changing?

0 = <9 years old

1 = 9‐10 years old

2 = 11‐12 years old

H9. Have you started to grow some hair on your face like a mustache or beard?

0 = No

1 = Yes

H10. "[If yes to above How old were you when you began growing a beard or mustache?

0 = <9 years old

1 = 9‐10 years old

2 = 11‐12 years old

**III. Identity**

Curiosity

*For each statement please answer with the following: Strongly disagree, disagree, agree or strongly agree.*

| **Characteristic** | | **Strongly disagree = 0** | **Disagree = 1** | **Agree = 2** | **Strongly Agree = 3** |
| --- | --- | --- | --- | --- | --- |
| CS1 | I like to think about problems and try to solve them in my head |  |  |  |  |
| CS2 | When I see a complex machine, I want to know how it works |  |  |  |  |
| CS3 | I would rather solve a problem myself than be told how to do it by someone else |  |  |  |  |
| CS4 | I like to observe things that are going on around me |  |  |  |  |
| CS5 | I like to learn new things |  |  |  |  |
| CS6 | When I don’t something, I try to learn more about it by asking my friends, family and or other adults |  |  |  |  |
| CS7 | It is interesting to handle rocks, sticks or seashells of different shapes and sizes |  |  |  |  |
| CS8 | I like to discover patterns in designs |  |  |  |  |
| CS9 | I like to sit quietly and listen to the birds sing and the cars pass by |  |  |  |  |
| CS10 | It’s fun to look at unusual works of art |  |  |  |  |

Purpose/Identity

*For each statement please answer with the following: Strongly disagree, disagree, agree, or strongly agree.*

| **Characteristic** | | **Strongly disagree = 0** | **Disagree = 1** | **Agree = 2** | **Strongly Agree = 3** |
| --- | --- | --- | --- | --- | --- |
| PID1 | I have decided on the direction I am going to follow in my life |  |  |  |  |
| PID2 | I have plans for what I am going to do in the future |  |  |  |  |
| PID3 | I think about different goals that I might pursue |  |  |  |  |
| PID4 | My plan for the future match with my true interests and values |  |  |  |  |
| PID5 | My future plans give me self confidence |  |  |  |  |
| PID6 | I talk with other people about the future plans I already made |  |  |  |  |

Persistence

*For each statement please answer with the following: Strongly disagree, disagree, agree or strongly agree.*

| **Characteristic** | | **Strongly disagree = 0** | **Disagree = 1** | **Agree = 2** | **Strongly Agree = 3** |
| --- | --- | --- | --- | --- | --- |
| PS1 | I usually persist in what I am doing |  |  |  |  |
| PS2 | Even if I fail to solve a problem, I try again and again and hope that I will find the solution |  |  |  |  |
| PS3 | When I read a book, I do not skip any pages |  |  |  |  |
| PS4 | When I do not understand something, I will ask my teacher again and again until I understand |  |  |  |  |
| PS5 | When I fail something, I am willing to try again and again forever |  |  |  |  |
| PS6 | I will continue with my hobby even though I haven’t had much success in it |  |  |  |  |
| PS7 | When I do not understand something, I usually ask for an explanation |  |  |  |  |
| PS8 | When I am in the classroom, I try to answer all the questions asked in the class |  |  |  |  |
| PS9 | I like to finish all my homework on time, even though sometimes it's hard and I'd rather go play |  |  |  |  |
| PS10 | If I'm not able to solve a problem, I keep trying until I find a solution or a different approach |  |  |  |  |

Generosity

*For each statement please answer with the following: Strongly disagree, disagree, agree or strongly agree.*

| **Characteristics** | | **Strongly disagree = 0** | **Disagree = 1** | **Agree = 2** | **Strongly Agree = 3** |
| --- | --- | --- | --- | --- | --- |
| GS1 | When one of my loved ones needs my attention, I really try to slow down and give them the time and help they need. |  |  |  |  |
| GS2 | I am known by family and friends as someone who makes time to pay attention to others’ problems. |  |  |  |  |
| GS3 | I’m the kind of person who is willing to go the “extra mile” to help take care of my friends,  relatives, and acquaintances. |  |  |  |  |
| GS4 | When friends or family members experience something upsetting or discouraging I make a special point of being kind to them. |  |  |  |  |
| GS5 | When it comes to my personal relationships with others, I am a very generous person. |  |  |  |  |
| GS6 | It makes me very happy to give to other people in ways that meet their needs. |  |  |  |  |
| GS7 | It is just as important to me that other people around me are happy and thriving as it is that I am happy and thriving. |  |  |  |  |
| GS8 | My decisions are often based on concern for the welfare of others. |  |  |  |  |
| GS9 | I am usually willing to risk my own feelings being hurt in the process if I stand a chance of  helping someone else in need. |  |  |  |  |
| GS10 | I make it a point to let my friends and family know how much I love and appreciate them. |  |  |  |  |
| UTU1 | I try and show Utu to other people |  |  |  |  |
| UTU2 | Being kind to other people helps me to show Utu |  |  |  |  |
| UTU3 | It makes me feel good when others show Utu |  |  |  |  |

**V. School**

SC1. How many days in a week are you given school work to do at home?

0 = None

1 = 1 day

2 = 2 days

3 = 3 days

4 = 4 days

5 = every day

SC2. Do you always have enough time to finish your homework on time?

0=No

1= Yes

____if no, why?

Household Chores

Do you help with any of the following household chores at home? If yes, how many hours per day do you spend on each chore?

|  | | No = 0 | Yes = 1 | Hours per day |
| --- | --- | --- | --- | --- |
| HC1 | Preparing food or cooking |  |  |  |
| HC2 | Washing dishes |  |  |  |
| HC3 | Cleaning the house and surroundings e.g. sweeping, mopping, throwing trash |  |  |  |
| HC4 | Washing clothes (laundry) |  |  |  |
| HC5 | Fetching water |  |  |  |
| HC6 | Taking care of younger siblings |  |  |  |

0 = None 1 = 1 hour 2 = 1-2 hours 3 = More than 2 hours

School climate perceptions.

*For each statement please answer with the following: Strongly disagree, disagree, agree or strongly disagree*

|  | **Characteristic** | **Strongly disagree = 0** | **Disagree = 1** | **Agree = 3** | **Strongly Agree = 4** |
| --- | --- | --- | --- | --- | --- |
| SCP1 | I feel close to my peers at school. |  |  |  |  |
| SCP2 | I am happy to be at school. |  |  |  |  |
| SCP3 | I feel proud to belong to my school. |  |  |  |  |
| SCP4 | The students at school are motivated to learn. |  |  |  |  |
| SCP5 | Teachers treat students fairly at school. |  |  |  |  |
| SCP6 | I feel safe at school. |  |  |  |  |
| SCP7 | I feel safe on my way to and from school. |  |  |  |  |
| SCP8 | I look forward to going to school |  |  |  |  |
| SCP9 | Students in my school respect each other |  |  |  |  |
| SCP10 | My parents or guardians are supportive of this school |  |  |  |  |
| SCP11 | I feel comfortable seeking help from my teachers when I need it |  |  |  |  |
| SCP12 | I am excited about the things I learn in school |  |  |  |  |

**V. Learning**

Team Skills Index

*For each statement please answer whether you do the following behavior when you work in a team at school with never, sometimes, often or always.*

| **Characteristic** | | **Never = 0** | **Sometimes = 1** | **Often = 2** | **Always = 3** |
| --- | --- | --- | --- | --- | --- |
| TS11 | I share ideas with others to accomplish a task |  |  |  |  |
| TSI2 | I enjoy helping team members |  |  |  |  |
| TSI3 | I value opinions that are different from my own |  |  |  |  |
| TSI4 | I cooperate with other students |  |  |  |  |
| TSI5 | I like team activities |  |  |  |  |
| TSI6 | I respect the opinion of my peers |  |  |  |  |
| TSI7 | I like to be in charge of groups or projects |  |  |  |  |
| TSI8 | When I have an idea, I speak up and share it with others |  |  |  |  |
| TSI9 | I believe I am a good leader |  |  |  |  |
| TSI10 | I suggest alternative solutions to problems |  |  |  |  |
| TSI11 | I am a good listener |  |  |  |  |
| TSI12 | I am willing to listen to listen and consider alternatives for handling a problem |  |  |  |  |
| TSI13 | I understand that each team member is different |  |  |  |  |

Growth Mindset/Outlook on Learning.

*For each statement please answer with the following: No, Neither Yes nor No, or Yes.*

| **Characteristic** | | **Strongly Disagree = 0** | **Disagree = 1** | **Agree = 2** | **Strongly Agree = 3** |
| --- | --- | --- | --- | --- | --- |
| OLN1 | I can do most things I try |  |  |  |  |
| OLN2 | I get excited when I learn something new |  |  |  |  |
| OLN3 | Challenges make me smarter |  |  |  |  |
| OLN4 | I can learn new things, but I can’t really change how intelligent I am |  |  |  |  |
| OLN5 | I like learning new things |  |  |  |  |
| OLN6 | I have a certain amount of intelligence and I really can’t do much to change it |  |  |  |  |
| OLN7 | My Intelligence is something that I can’t change very much |  |  |  |  |
| OLN8 | No matter who I am, I can significantly change my intelligence level |  |  |  |  |
| OLN9 | The harder I work at something, the better I will be at it |  |  |  |  |

Goal Orientation Scale

*For each statement please answer with the following: Strongly disagree, disagree, agree, or strongly agree.*

| **Characteristic** | | **Strongly disagree = 0** | **Disagree = 1** | **Agree = 3** | **Strongly Agree = 4** |
| --- | --- | --- | --- | --- | --- |
| GO1. | I want to do well at school to show that I can learn new things |  |  |  |  |
| GO2. | I want to do well in school because being better than others is important to me |  |  |  |  |
| GO3. | I choose easy options in school so that I don’t have to work too hard |  |  |  |  |
| GO4. | I want to do well at school so that I can feel close to my group of friends |  |  |  |  |
| GO5. | I want to do well at school so that I can get praise from my teachers |  |  |  |  |
| GO6. | I try to do well at school so that I can help my friends with their school work when they need it |  |  |  |  |
| GO7. | I want to do school work because other people expect it of me |  |  |  |  |
| GO8. | I do schoolwork so that I can get a good job in the future |  |  |  |  |
| GO9. | When learning things for school, I try to see how they fit together with other things I already know |  |  |  |  |
| GO10. | I try to organize my school notes when I want to learn things for school |  |  |  |  |
| GO11. | When I want to learn things for school, I practice repeating them to myself |  |  |  |  |
| GO12. | I often ask myself questions to see if I understand what I am learning |  |  |  |  |
| GO13. | I often look through books to see how they are arranged before I start reading |  |  |  |  |
| GO14. | If I don’t understand my schoolwork, I ask the teacher to help me |  |  |  |  |

**VI. Social Relationships**

Family Expectations

*For each statement please answer with the following: No, Neither Yes nor No, or Yes.*

| **Characteristic** | | **No = 0** | **Neither yes nor no = 1** | **Yes= 2** |
| --- | --- | --- | --- | --- |
| FEX1. | My family expects me to have good grades. |  |  |  |
| FEX2. | My family expects me to graduate primary school and go on to secondary school |  |  |  |
| FEX3. | My family expects me to graduate from high school. |  |  |  |
| FEX4. | My family expects me to go to university. |  |  |  |
| FEX5. | I can talk to my family about my worries or concerns |  |  |  |
| FEX6. | I can talk to my family about changes in my body |  |  |  |
| FEX7. | My family cares about what I am thinking and feeling |  |  |  |
| FEX8. | My family usually knows where I am |  |  |  |
| FEX9. | My family is accepting of me having a boyfriend or girlfriend at this time in my life |  |  |  |

Peer Expectations

*For each statement please answer with the following: Never, Sometimes, Often, or Always*

| **Characteristic** | | **Never = 0** | **Sometimes = 1** | **Often = 2** | **Always = 3** |
| --- | --- | --- | --- | --- | --- |
| PEX1. | My close friends think it’s important to attend school regularly. |  |  |  |  |
| PEX2. | My close friends think it’s important to study hard. |  |  |  |  |
| PEX3. | My close friends think it’s important to be good in sports. |  |  |  |  |
| PEX4. | My close friends think it’s important to be popular with people my age |  |  |  |  |
| PEX5. | My close friends think it’s important that I pay attention to my appearance |  |  |  |  |
| PEX6. | My close friends think it’s important to have a boyfriend or girlfriend |  |  |  |  |

Bullying:

*For each statement please answer with the following: Yes or No*

| **Characteristic** | | **No = 0** | **Yes = 1** |
| --- | --- | --- | --- |
| BU1 | Have you ever been bullied? |  |  |
| BU2 | In the past 30 days, were you hit, kicked, pushed, shoved around or locked in doors? |  |  |
| BU3 | In the past 30 days, were you made fun of because of your religion? |  |  |
| BU4 | In the past 30 days, were you made fun of with sexual jokes, comments or gestures? |  |  |
| BU5 | In the past 30 days, were you left out of activities on purpose or completely ignored |  |  |
| BU6 | In the past 30 days, were you made fun of because of how your body or face looks? |  |  |
| BU7 | In the past 30 days, were you bullied in some other way? |  |  |

**VII. Mental Health and Well-being**

Psychosocial Assessment

*For each statement please answer with the following: Never, Somewhat, Often, or All the time.*

| **Characteristic** | | **Never = 0** | **Somewhat = 1** | **Often = 2** | **All the time = 3** |
| --- | --- | --- | --- | --- | --- |
| PA 1 | I listen to others |  |  |  |  |
| PA 2 | I play together with others |  |  |  |  |
| PA 3 | I have a lot of thoughts |  |  |  |  |
| PA 4 | I worry constantly |  |  |  |  |
| PA 5 | I think I am of no use |  |  |  |  |
| PA 6 | I think about suicide |  |  |  |  |
| PA 7 | I sit alone |  |  |  |  |
| PA 8 | I share with others |  |  |  |  |
| PA 9 | I feel a lot of pain in my heart |  |  |  |  |
| PA 10 | I sit with my cheek in my palm |  |  |  |  |
| PA 11 | I cry when I am alone |  |  |  |  |
| PA 12 | I do not sleep at night |  |  |  |  |
| PA 13 | I am disobedient |  |  |  |  |
| PA 14 | I share food and eat with others |  |  |  |  |
| PA 15 | I have a lot of worries |  |  |  |  |
| PA 16 | I want to be alone |  |  |  |  |
| PA 17 | I hold my head |  |  |  |  |
| PA 18 | I drink alcohol |  |  |  |  |
| PA 19 | I insult friends |  |  |  |  |
| PA 20 | I help others |  |  |  |  |
| PA 21 | I don’t think straight |  |  |  |  |
| PA 22 | I mutter to myself |  |  |  |  |
| PA 23 | I feel I can do nothing to help myself |  |  |  |  |
| PA 24 | I fight |  |  |  |  |
| PA 25 | I use bad language |  |  |  |  |
| PA 26 | I am disrespectful |  |  |  |  |
| PA 27 | I misbehave |  |  |  |  |
| PA 28 | I welcome others |  |  |  |  |
| PA 29 | I deceive |  |  |  |  |
| PA 30 | I am a rough person |  |  |  |  |
| PA 31 | I use drugs |  |  |  |  |
| PA 32 | I think people are chasing me |  |  |  |  |
| PA 33 | I cooperate with others |  |  |  |  |
| PA 34 | I feel sad |  |  |  |  |
| PA 35 | I think of bad things |  |  |  |  |
| PA 36 | I cry continuously |  |  |  |  |
| PA 37 | I respect others |  |  |  |  |

Empathy

*For each statement please answer with the following: Not True, Sometimes True, Often True*

| **Characteristic** | | **Not True = 0** | **Sometimes True= 1** | **Often True = 2** |
| --- | --- | --- | --- | --- |
| EMP1 | If my parent/guardian is happy, I also feel happy |  |  |  |
| EMP2 | I often feel sad when I watch a sad movie |  |  |  |
| EMP3 | When a friend is upset, I feel upset too |  |  |  |
| EMP4 | When a friend cries, I cry myself |  |  |  |
| EMP5 | If someone in my family is sad, I feel really bad |  |  |  |
| EMP6 | I feel awful when two people quarrel |  |  |  |
| EMP7 | When a friend is angry, I tend to know why |  |  |  |
| EMP8 | If a friend is sad, I understand mostly why |  |  |  |
| EMP9 | If a friend cries, I often understand what has happened |  |  |  |
| EMP10 | If a friend is sad, I like to comfort him/her |  |  |  |
| EMP11 | I would like to help when a friend gets angry |  |  |  |
| EMP12 | If a friend has an argument, I try to help |  |  |  |
| EMP13 | I want everyone to feel good |  |  |  |
| EMP14 | If a friend is sad, I want to do something to make it better |  |  |  |

**VIII. Empowerment**

Gender perceptions.

*For the following questions, please choose one of the following: Disagree or Agree*

| **Characteristic** | | **Disagree = 0** | **Agree = 1** |
| --- | --- | --- | --- |
| GEM1. | Girls are better than boys at learning new things |  |  |
| GEM2. | Boys are better than girls at solving problems |  |  |
| GEM3. | In team activities, I work best with kids my own gender |  |  |
| GEM4. | Boys are better at achieving their goals than girls |  |  |
| GEM5. | Girls should have time to study after school |  |  |
| GEM6. | Girls should have time to play with friends after school |  |  |
| GEM7 | Boys and girls should be equally responsible for doing household chores |  |  |
| GEM8 | I prefer playing games with kids my own gender |  |  |
| GEM9 | I prefer studying or doing homework with kids my own gender |  |  |
| GEM10 | It is more important for a girl to help at home and learn household activities than to spend time studying |  |  |
| GEM11 | Boys should have more free time overall than girls |  |  |
| GEM12 | It is important for a man to act tough/strong |  |  |
| GEM13 | Certain careers/jobs such as police officer, engineering, military police are only suitable for men |  |  |

Self-Efficacy

*For each statement please answer with the following: Not at all, a little bit, about average, well, or very well.*

| **Characteristic** | | **Not at all = 0** | **A little bit = 1** | **About Average= 2** | **Well = 3** | **Very well = 4** |
| --- | --- | --- | --- | --- | --- | --- |
| SEQ1. | I can ask teachers to help me when I get stuck on schoolwork. |  |  |  |  |  |
| SEQ2. | I can express my opinions when others disagree with me. |  |  |  |  |  |
| SEQ3. | I can succeed at cheering myself up when something bad has happened to me. |  |  |  |  |  |
| SEQ4. | I can study even if there are other interesting things to do. |  |  |  |  |  |
| SEQ5. | I can succeed in being calm even when I'm scared. |  |  |  |  |  |
| SEQ6. | I can become friends with other children. |  |  |  |  |  |
| SEQ7. | I can study for a test. |  |  |  |  |  |
| SEQ8. | I can chat with an unfamiliar person. |  |  |  |  |  |
| SEQ9. | I can stop myself from being nervous. |  |  |  |  |  |
| SEQ10. | I can finish my homework every day. |  |  |  |  |  |
| SEQ11. | I can work in harmony with my classmates. |  |  |  |  |  |
| SEQ12. | I can control my feelings. |  |  |  |  |  |
| SEQ13. | I can pay attention during every class. |  |  |  |  |  |
| SEQ14. | I can tell other children that they are doing something I don't like. |  |  |  |  |  |
| SEQ15. | I can give myself a pep talk when I feel low. |  |  |  |  |  |
| SEQ16. | I can succeed in understanding all subjects in school. |  |  |  |  |  |
| SEQ17. | I can tell a funny event to a group of children. |  |  |  |  |  |
| SEQ18. | I can tell a friend if I don't feel well. |  |  |  |  |  |
| SEQ19. | I succeed in making my parents satisfied with my schoolwork. |  |  |  |  |  |
| SEQ20. | I can succeed in staying friends with other children. |  |  |  |  |  |
| SEQ21. | I can succeed in stopping unpleasant thoughts. |  |  |  |  |  |
| SEQ22. | I can succeed in passing tests at school. |  |  |  |  |  |
| SEQ23. | I can prevent arguments with other children. |  |  |  |  |  |
| SEQ24. | I can succeed in not worrying that bad things might happen. |  |  |  |  |  |
| SEQ25. | I feel like I have control over making decisions that affect my everyday activities. |  |  |  |  |  |
| SEQ26. | I feel like I have a big impact in making this neighborhood a better place to live |  |  |  |  |  |

**IX. Technology**

The following questions are about your access to and use of media, for example: TV, radio, movies, computers, Internet, mobile phones.

*For each item, please tell me if you have access to the following forms of technology.*

| **Characteristic** | | **No, do not have = 0** | **Yes, but don’t have my own = 1** | **Yes, I have my own= 2** |
| --- | --- | --- | --- | --- |
| T1. | Television |  |  |  |
| T2. | Radio |  |  |  |
| T3. | Computer/laptop /tablet (eg. iPad) with internet connection |  |  |  |
| T4. | Cell/mobile phone |  |  |  |
| T5. | Smart phone with internet connection |  |  |  |

T5. On a typical school day, how many hours total do you spend using the following technology?

| **Technology** | | **None = 0** | **About 1 hour = 1** | **About 2 hours= 2** | **More than 2 hours=3** |
| --- | --- | --- | --- | --- | --- |
| T6. | Watching Television/Movies |  |  |  |  |
| T7. | Listening to the Radio |  |  |  |  |
| T8. | Using the internet on a computer/laptop/tablet |  |  |  |  |
| T9. | Talking or chatting with friends on a mobile phone |  |  |  |  |
| T10. | Using other media |  |  |  |  |

Technology Perceptions.

*For each statement please answer with the following: Never, Sometimes, Often, Always*.

| **Characteristic** | | **Never = 0** | **Sometimes = 1** | **Often= 2** | **Always = 3** |
| --- | --- | --- | --- | --- | --- |
| TP1. | Technology will provide solutions to many of our problems. |  |  |  |  |
| TP2. | With technology anything is possible. |  |  |  |  |
| TP3. | Technology makes people waste too much time. |  |  |  |  |
| TP4. | Technology makes life more complicated. |  |  |  |  |
| TP5. | I feel that computers help me learn new things. |  |  |  |  |
| TP6. | I feel that tablets help me learn new things. |  |  |  |  |
| TP7. | I feel that the internet helps me learn new things. |  |  |  |  |
| TP8. | I feel confident using a computer/tablet. |  |  |  |  |
| TP9. | I feel confident showing someone else how to use a computer/tablet. |  |  |  |  |

**X. Distress Screening**

DS1. I know the some of the questions that I asked may have been sensitive or uncomfortable for you to talk about. Can you tell me how are you feeling right now?

0 = Good (happy, not at all upset)

1 = Ok (nor happy or upset)

2 = Somewhat worried/upset

3 = Very worried/upset

4 = Reported abuse

DS2.If you are comfortable telling me, please tell me what has upset or worried you?

If the respondent reports abuse or that they are very worried/upset:

*“Based on your saying to me [or showing] that our interview may have upset you, I would like to share this with my supervisor [field coordinator] so that we can let you know where to find help that might be useful. If ok with you, we will also talk to your mother (or father) so that they can help you”*

[If the adolescent does not want to share this with his/her parents/guardians, help them identify another adult they could talk with].

If the respondent does not report abuse, and is not very worried/upset:

*“We have talked about many things today that you might have more questions about. I want to give you this card with numbers and locations for organizations [say the local names] that work with young people your age. You might have heard of some, and some might be new to you. If you have questions or want to talk to someone, you can call them and they will try to help you.”*

**XI. Interviewer Assessment**

Interviewer, please complete the questions below based on your own observation and assessment of the entire interview process, and the respondent.

IA1. How did you find the respondent’s cooperation?

0 = Very good

1 = Moderate (ok)

2 = Bad

3 = Very bad

If bad or very bad, please explain why:

IA2.How accurate/true did you find the respondent’s answers?

0 = Very accurate/true

1 = Somewhat accurate/true

2 = Not very accurate/true

3 = Highly inaccurate (the responses should not be trusted)"

If inaccurate, please explain why can’t be trusted:

IA3. How did you find the respondent’s understanding of the questions discussed?

0 = Very good (understood perfectly)

1 = Moderate (understood ok)

2 = Bad (did not understand many of the questions)

3= Very bad (did not understand at all)

If bad or very bad, please explain about their not understanding:

IA4. How did you find the respondent’s concentration and attentiveness during the interview?

0 = Very good (highly concentrated/attentive)

1 = Moderate/ok (somewhat concentrated/attentive)

2 = Bad (could not concentrate for many parts of the interview)

3= Very bad (could not concentrate at all)"

I bad or very bad please explain why very bad at concentration:

IA5. About how many breaks did you take during the full interview?

NUMERICAL.

**Scale for Measuring Level of Engagement with Parent-Youth Workbook**

|  | **Survey Question** | **Scale** |
| --- | --- | --- |
| PW1 | While at home, do you use the parent workbook with your parent/guardian? | No =0  Yes =1 |
| PW2 | In the past one month, who has been using the workbook with you at home, the most? | Mother =1  Father =2  Sibling =3  Friend =4  Other family member:  (Please specify) =5  No one =6 |
| PW3 | How many days a week do you usually use the workbook with your parent/guardian? | 0 days =1  1-2 days =2  3-4 days =3  5-6 days =4  Everyday =5 |
| PW4 | Do you find the workbook activities interesting? | No =1  Some of the time =2  Most of the time =3  All of the time =4 |
| PW5 | What component of the workbook do you find most interesting? | Writing assignment =1  Discussion with parent =2  Activity with parent =3  Gratitude Jar Activity =4 |
| PW6 | Does your parent/guardian listen when you have something to say during the workbook sessions? | No =1  Some of the time =2  Most of the time =3  All of the time =4 |
| PW7 | Do you finish all the workbook’s assignments? | No =1  Some of the time =2  Most of the time =3  All of the time =4 |
| PW8 | In the past one-month, how much did you remind your parent/guardian to use the workbook? | None of the time =1  Some of the Time =2  Most of the Time =3  All lot of the time =4 |
| PW9 | In the past one-month, how much has your parent/guardian reminded you to use the workbook? | None of the time =1  Some of the Time =2  Most of the Time =3  All lot of the time =4 |
| *For each statement please answer with the following: Strongly disagree, disagree, agree or strongly disagree* | | |
| PW10 | I enjoy using the parent workbook with my parent/guardian | Strongly disagree =1  Disagree =2  Agree =3  Strongly agree =4 |
| PW11 | Working on the workbook together has helped me foster a stronger relationship with my parent/guardian | Strongly disagree =1  Disagree =2  Agree =3  Strongly agree =4 |
| PW12 | Working on the workbook together has helped my family appreciate equal opportunities for boys and girls | Strongly disagree =1  Disagree =2  Agree =3  Strongly agree =4 |
